# Axial Chirality Induction in Ferrocene-Amino Acid Hybrids—toward Chiral Redox-Active Building Blocks

**DOI:** 10.1021/acs.inorgchem.6c01109

**Published:** 2026-05-04

**Authors:** Marcin Konopka, Rafał Śliwa, Marek P. Szymański, Aleksandra Tobolska, Wojciech Wróblewski, Ireneusz Tomczyk, Katarzyna Rybicka-Jasińska, Artur Kasprzak, Agnieszka Szumna

**Affiliations:** † Institute of Organic Chemistry, Polish Academy of Sciences, Kasprzaka 44/52, 01-224 Warsaw, Poland; ‡ Faculty of Chemistry, 201870 Warsaw University of Technology, Noakowskiego 3, 00-664 Warsaw, Poland

## Abstract

In this paper, we report the construction of chiral ferrocene-containing (Fc) supramolecular platforms with high symmetry and well-defined geometry via dynamic covalent chemistry. Starting from amino acid hydrazides and ferrocene aldehydes, we obtained respective Fc-acylhydrazones with well-defined conformations, that display intense circular dichroism (CD) signals in the visible region associated with ferrocene-centered electronic transitions. CD and UV–vis spectroscopy, supported by computational analysis, reveal efficient transfer of chirality from the remote stereogenic centers to the Fc unit and a correlation between amino acid configuration, Fc helicity, and CD sign (l-amino acid → (*P*)-Fc → (−)­CD at 470 nm). Exploiting the dynamic character of the acylhydrazone linker, amino acid trihydrazide **l** **-2** was further assembled with ferrocene dialdehyde into *D*_3_-symmetric double-decker trigonal platform **Fc**_**3**_ **(** **l** **-2)**_**2**_ with high synthetic efficiency (>90%). Under these conditions, narcissistic chiral self-sorting occurs via dynamic covalent exchange, which results in the formation of homochiral platforms from racemic precursors. The combination of conformationally locked axial chirality, reversible covalent connectivity, and inherent redox activity establishes new amino acid–based motifs as promising modules for constructing chiral Fc-containing porous architectures such as molecular cages, MOFs, and COFs, with potential for asymmetric electrochemical applications.

## Graphical abstract

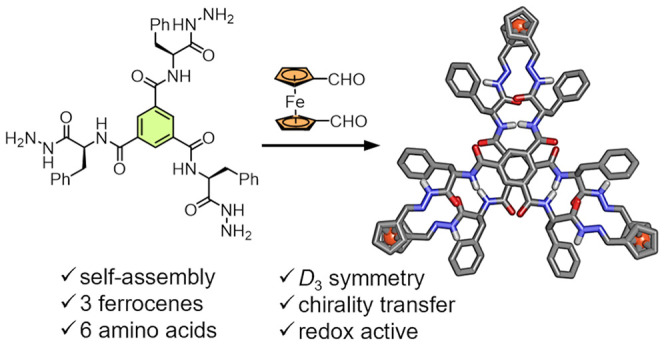

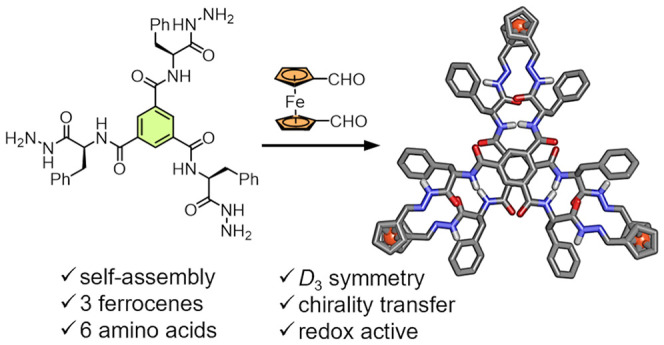

## Introduction

Ferrocene (Fc) units incorporated into molecular architectures can perform in a wide range of functions. Owing to their capacity for uniaxial intramolecular rotation^1,2^ and the existence of two readily switchable redox states (Fc^+^/Fc, 0.64 V vs SHE)^3^ with distinct spin, charge, optical, and chemical properties, ferrocene units can serve as redox switches,^4,5^ catalysts,^6^ sensors,^7,8^ components of spintronic devices,^9,10^ or pivoting elements.^11^ Fc-functionalized molecular cages^12–14^ and intrinsically porous frameworks (e.g., MOFs and COFs)^15^ combine the redox activity of the electron-rich ferrocene unit with the encapsulation and sorption capabilities of porous scaffolds. As a result, these hybrid systems exhibit enhanced performance in selective oxidations and coupling reactions,^16,17^ electrochemical sensing,^18^ energy storage^19^ and conversion processes (e.g., in batteries and supercapacitors).

Despite substantial progress in the design and fabrication of Fc-containing porous structures, significant challenges remain. A key limitation is the synthetic accessibility of Fc-based building blocks that combine the required rigidity, symmetry, and functionalization potential, ideally assembled via reversible reactions. An additional constraint, impeding progress toward asymmetric applications such as asymmetric catalysis,^20^ enantiomer separation, and chiral sensing, arises from the limited availability of chiral Fc-containing building blocks. This situation comes primarily from (1) the difficulty of inducing and locking axial chirality at the Fc unit and/or (2) the intrinsic conformational lability of chiral substituents bearing sp^3^-stereogenic centers. As a consequence of this high flexibility, most Fc-based chiral fragments lack shape persistence, making them poorly suited for the construction of robust porous architectures.

In this work, we introduce a strategy based on dynamic covalent chemistry (DCvC) to access chiral Fc-based building blocks that combine high symmetry, well-defined geometry, and redox activity. Amino acids, readily available multifunctional chiral resources, are first converted into hydrazide derivatives and then transformed into robust scaffolds that induce conformational axial chirality at the Fc unit. We demonstrate the stereoselective formation of *C*_2_-symmetric linear building blocks and *D*_3_-symmetric double-decker architectures, which have high stability and retain the structural motif of axially chiral Fc-unit (also achievable via homochiral narcissistic self-sorting). These building blocks arise from reversible acylhydrazone formation, making them particularly suitable for constructing porous structures by dynamic covalent chemistry. Notably, reversible linkages such as imines^21^ and boronate esters^22^ have been previously employed to incorporate Fc units into trigonal architectures (Figure 1); however, acylhydrazone linkers have not been used in this context, and only achiral structures of this general type have been described to date.

**Figure 1:**
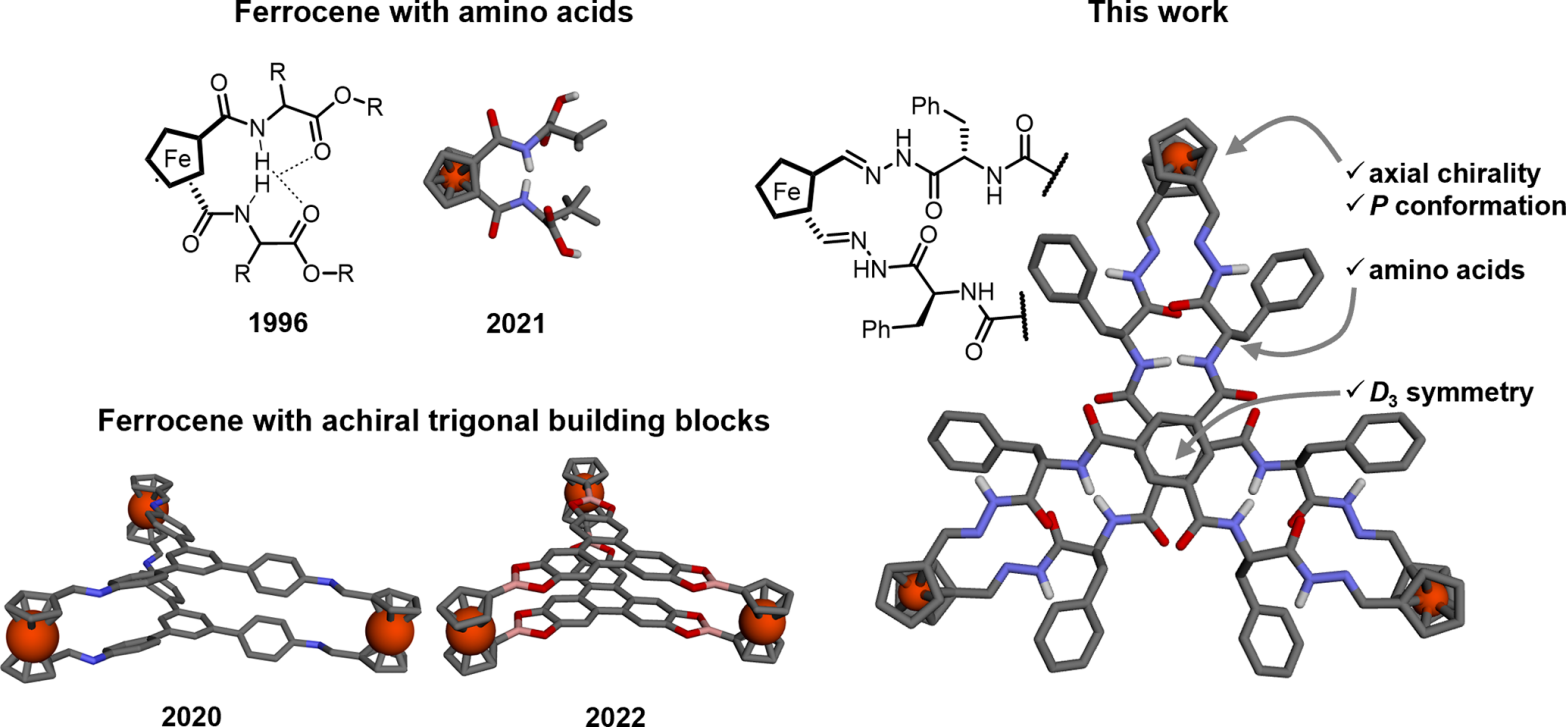
Previously reported supramolecular architectures containing ferrocene motifs (refs 12, 22, 24 and 25 ) and the tripodal double-decker structure described in this work.

## Results and Discussion

### Design and Synthesis

Cyclopentadienyl (Cp) rings in Fc units can be readily functionalized (e.g., with carboxylic acid or amino groups), providing handles for further modification with chiral derivatives. Amino acids and peptides are particularly valuable chiral fragments to attach to Fc units because, when brought into close proximity, they can engage in the noncovalent interactions between appended strands, transfer their chirality onto the Fc core, and rigidify the overall structure. Numerous peptide–Fc hybrids have been synthesized and analyzed to identify predictable structural motifs.^23^ In 1996, Herrick and co-workers showed that, in nonpolar solvents, ferrocene dicarboxylic acid derivatives functionalized with amino acids or peptides form a pair of intramolecular hydrogen bonds and adopt a *C*_2_-symmetric conformation, accompanied by chirality transfer to the Fc unit (Figure 1).^24,25^ Subsequent studies confirmed the generality of this motif^26^ and established correlations between amino acid configuration, Fc helicity, and CD response (l-AA → (*P*)-Fc → (+)­CD at 470 nm). This Herrick-type motif has since been exploited to direct self-assembly,^27^ construct complex chiral macrocycles,^28^ and develop chiroptical switching.^25,29^ Our group has a long-standing interest in harnessing amino acids and short peptides to form highly ordered assemblies, including porous cages.^30–32^ We have found that *C*-terminus-modified peptides bearing hydrazide groups offer an advantageous balance of stability, reversibility, and semirigidity for creating ordered architectures via dynamic covalent chemistry.^33^ In this work, we employ acylhydrazone’s formation to construct a new motif **Fc­(** **l** **-1)**_**2**_ that uses amino acid chirality to induce axial chirality at the Fc unit, and we show that multiple Fc–acylhydrazone–amino acid hybrids can be convergently connected to a central core to afford a robust, high-symmetry double-decker building block, **Fc**_**3**_ **(** **l** **-2)**_**2**_.

For the synthesis of chiral Fc–amino acid hybrids, l-phenylalanine (l-Phe) was chosen as a chiral source because it is known to enhance the solubility of supramolecular assemblies in nonpolar solvents.^34,35^ For the construction of trigonal building blocks, we selected a 1,3,5-trisubstituted benzene core, the smallest member of a broad family of trigonal aromatic scaffolds that serve as fundamental units for the assembly of cages and MOFs. Starting hydrazides **l** **-1**, **l** **-2,** and **d** **-2** were prepared according to known procedures.^36^ For **l** **-2**, benzene-1,3,5-tricarboxylic acid (BTA) was initially coupled with three equivalents of l-phenylalanine methyl ester hydrochloride using a standard 1-ethyl-3-(3-(dimethylamino)­propyl)­carbodiimide hydrochloride (EDC·HCl)-mediated condensation in DMF. The resulting tris-substituted BTA derivative was then treated with an excess of hydrazine hydrate in MeOH to give the tris-hydrazide **l** **-2** (see experimental section for synthetic protocols). **l** **-2** contains three stereogenic centers in the l-phenylalanine units (*S*,*S*,*S* absolute configuration) and inherits *C*_3_ symmetry from the BTA core, as confirmed by ^1^H NMR spectroscopy.

The Fc-containing derivatives **Fc­(** **l** **-1)**_**2,**_ **Fc**_**3**_ **(** **l** **-2)**_**2**_, and **Fc**_**3**_ **(** **l** **-2)** were obtained in one-pot condensations of **l** **-1** or **l** **-2** (10 mM) with stoichiometric amounts of ferrocenecarboxaldehyde (Fc­(CHO)) or 1,1′-ferrocenedicarboxaldehyde (Fc(CHO)_2_), carried out in THF at room temperature overnight (Figure 2a). After workup involving solvent removal and washing with MeCN, the products were isolated as dark-brown solids, typically in >90% yield (see Supporting Information for details). Their formation has been confirmed by 1D and 2D NMR spectroscopy and ESI-MS, which showed *m*/*z* signals corresponding to the [M – H]^−^ and [M – 2H]^2–^ ions with the expected isotopic patterns (see Supporting Information, Figure S14). Notably, the synthesis of **Fc**_**3**_ **(** **l** **-2)**_**2**_ requires the formation of six acylhydrazone bonds and the convergence of five building blocks; nevertheless, the transformation is highly efficient, and the ^1^H NMR spectrum in THF-*d*_8_ indicates a highly ordered structure with *D*_3_ symmetry (Figure 2c). ^1^H DOSY NMR analysis of **Fc**_**3**_ **(** **l** **-2)**_**2**_ has revealed discrete species with a hydrodynamic radius of ca. 10 Å (diameter ca. 20 Å, Figure S12). This is consistent with the dimensions in calculated model of **Fc**_**3**_ **(** **l** **-2)**_**2**_ (Figure 5f).

**Figure 2:**
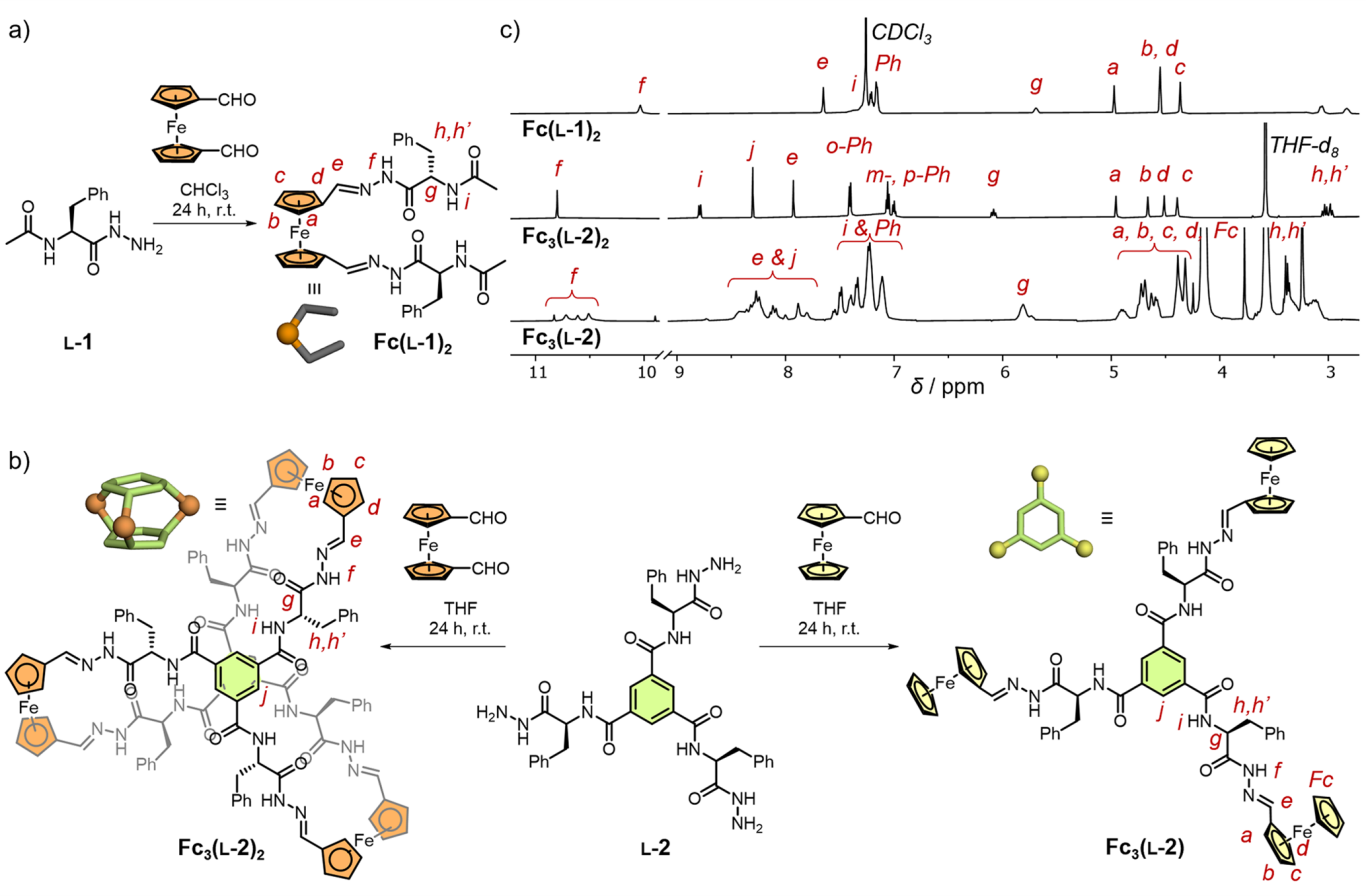
Synthesis of chiral ferrocene structures: (a) **Fc­(** **l** **-1)**_**2**_; (b) **Fc**_**3**_ **(** **l** **-2)**_**2**_ and **Fc**_**3**_ **(** **l** **-2)**; and (c) comparison of ^1^H NMR spectra of these compounds with signals assignments.

^1^H NMR spectra of **Fc­(** **l** **-1)**_**2**_ and **Fc**_**3**_ **(** **l** **-2)**_**2**_, which contain doubly substituted Fc units, are sharp and symmetry-averaged, indicating the formation of well-ordered structures (*C*_2_ and *D*_3_ symmetry, respectively; Figure 2c). In contrast, the spectrum of **Fc**_**3**_ **(** **l** **-2)** is markedly broadened, suggesting an ill-defined dynamic ensemble and/or the presence of multiple conformations. Such unsymmetrical features and broad resonances are typical for species bearing several acylhydrazone units, owing to *E*/*Z* isomerism.^37,38^ Thus, in **Fc­(** **l** **-1)**_**2**_ and **Fc**_**3**_ **(** **l** **-2)**_**2**_, acylhydrazone *E*/*Z* isomerization appears to be effectively suppressed, in contrast to **Fc**_**3**_ **(** **l** **-2)**. Moreover, the Fc’s Cp rings resonances in **Fc­(** **l** **-1)**_**2**_ and **Fc**_**3**_ **(** **l** **-2)**_**2**_ are split into four broad singlets (arising from the two doublets observed for the precursor Fc­(CHO)_2_), indicating that the Fc fragments adopt chiral conformations. Pronounced differentiation of the phenyl signals of the phenylalanine residues is observed for **Fc**_**3**_ **(** **l** **-2)**_**2**_, consistent with conformational fixation of these groups in **Fc­(** **l** **-1)**_**2**_, whereas such differentiation is absent for **Fc**_**3**_ **(** **l** **-2)**. This interpretation is supported by the ^1^H–^1^H ROESY spectrum, which shows through–space correlations between the ortho protons of the phenylalanine rings, the BTA core protons, and the terminal Fc protons. It is also noteworthy that the α-CH signal is shifted unusually far downfield (6.08 ppm, Δδ ≈ +1.3 ppm relative to **l** **-2**), consistent with its location in a deshielding environment.

### Chiral Self-Sorting

The well-defined multicomponent structure of **Fc**_**3**_ **(** **l** **-2)**_**2**_, together with the reversible nature of the hydrazone bonds formed during its synthesis, prompted us to investigate the possibility of chiral self-sorting. Dialdehyde Fc­(CHO)_2_ (2 equiv.) was reacted with an equimolar mixture of **l** **-2** and **d** **-2** (1 equiv. each; total concentration 10 mM in THF-*d*_8_). The resulting ^1^H NMR spectrum of the reaction mixture was nearly identical to that obtained for the reactions with the enantiomerically pure ligands, and no additional signals indicative of mixed assemblies **Fc**_**3**_ **(** **l** **-2)­(** **d** **-2)** or other side products were detected in the ^1^H NMR spectrum, consistent with quantitative self-sorting (Figure 3). The observed narcissistic chiral self-sorting demonstrates a pronounced preference for the formation of homochiral architecture and further underscores the robustness of the assembly.

**Figure 3:**
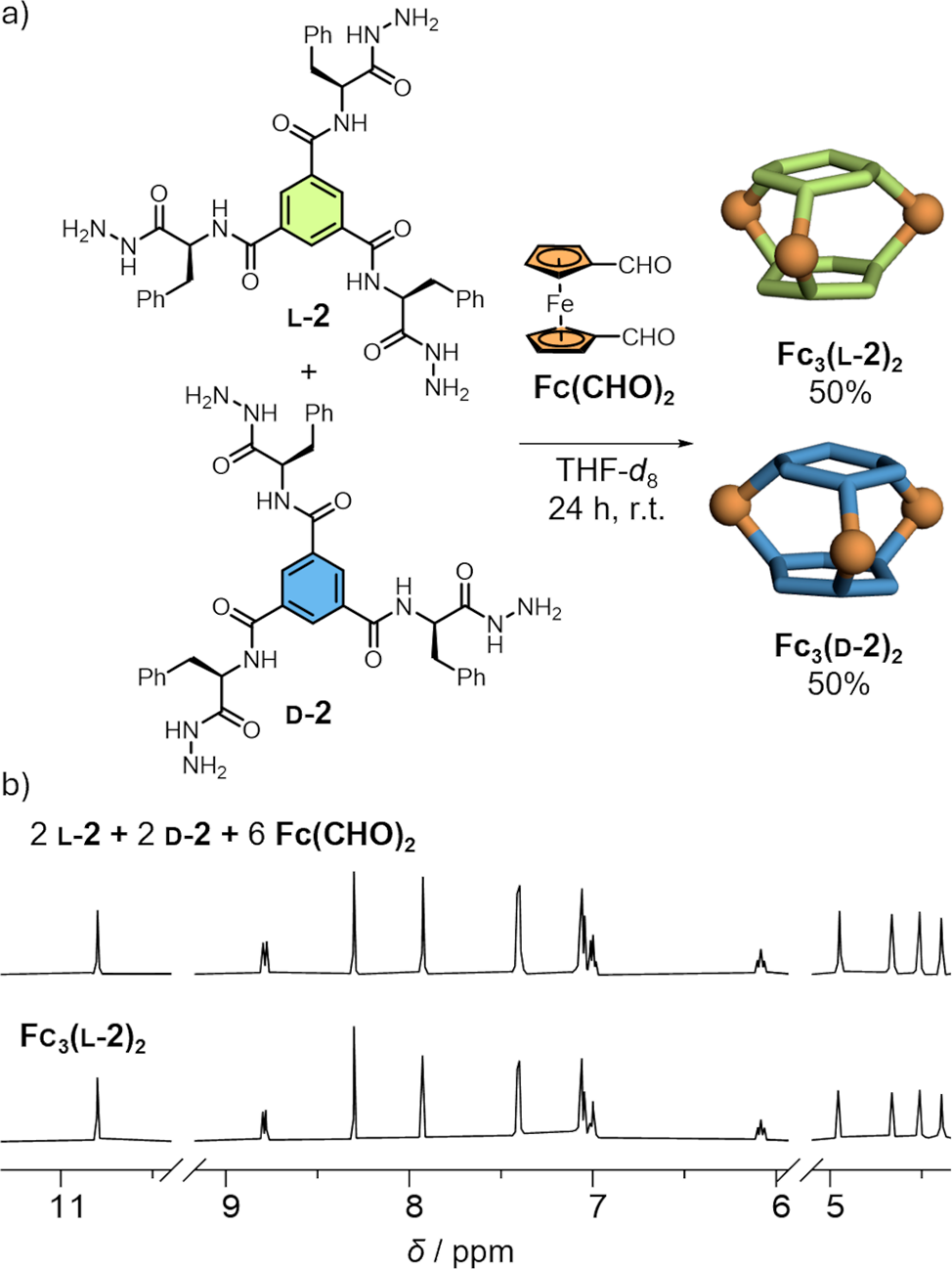
(a) Self-sorting experiment; (b) comparison of ^1^H NMR spectra between postreaction mixture and pure **Fc**_**3**_ **(** **l** **-2)**_**2**_ (see Supporting Information, Figure S15 for **Fc**_**3**_ **(** **d** **-2)**_**2**_ spectrum).

### CD Spectra and Theoretical Calculations

Chiroptical properties of all products were determined by circular dichroism (CD) spectroscopy in solvents of differing polarity and hydrogen-bonding ability (DCM, THF, DMF, Figure 4). For the disubstituted Fc derivatives **Fc­(** **l** **-1)**_**2**_ and **Fc**_**3**_ **(** **l** **-2)**_**2**_, pronounced CD signals are observed for the Fc-centered d–d transitions (ca. 440 and 325 nm) in noncompetitive solvents (DCM and THF), indicating efficient transfer of chirality from the amino acid–based stereogenic centers to the Fc units. In both cases, the l configuration of the amino acid gives rise to negative CD bands at 440 and 325 nm. Notably, the magnitude of the effect per Fc unit (Δε/number of Fc units) is the same for **Fc­(** **l** **-1)**_**2**_ and **Fc**_**3**_ **(** **l** **-2)**_**2**_, suggesting that the chiral environments present in monomeric unit **Fc­(** **l** **-1)**_**2**_ and in rigidified cage-like structure of **Fc**_**3**_ **(** **l** **-2)**_**2**_ are similar in noncompetitive solvents. In DMF, a more competitive solvent, the extent of chirality transfer is substantially reduced for **Fc­(** **l** **-1)**_**2**_, whereas for **Fc**_**3**_ **(** **l** **-2)**_**2**_ it is much less affected, consistent with its more rigid structure. Comparison of the Fc-centered CD bands for **Fc­(** **l** **-1)**_**2**_ and **Fc**_**3**_ **(** **l** **-2)**_**2**_ (Δε ≈ −5.0 M^–1^ cm^–1^) with literature data for axially chiral Herrick-type derivatives (Δε ≈ +1.2 M^–1^ cm^–1^)^27^ reveals that they have the opposite signs, but their intensities are of the same order of magnitude (or even larger in our case). This is a remarkable observation given that for **Fc­(** **l** **-1)**_**2**_ and **Fc**_**3**_ **(** **l** **-2)**_**2,**_ the stereogenic centers are separated from the Fc units by five mostly planar bonds. This demonstrates that the acylhydrazone linker is highly effective in transmitting the amino acid chirality into the axially chiral, twisted conformation of the Fc units.

**Figure 4:**
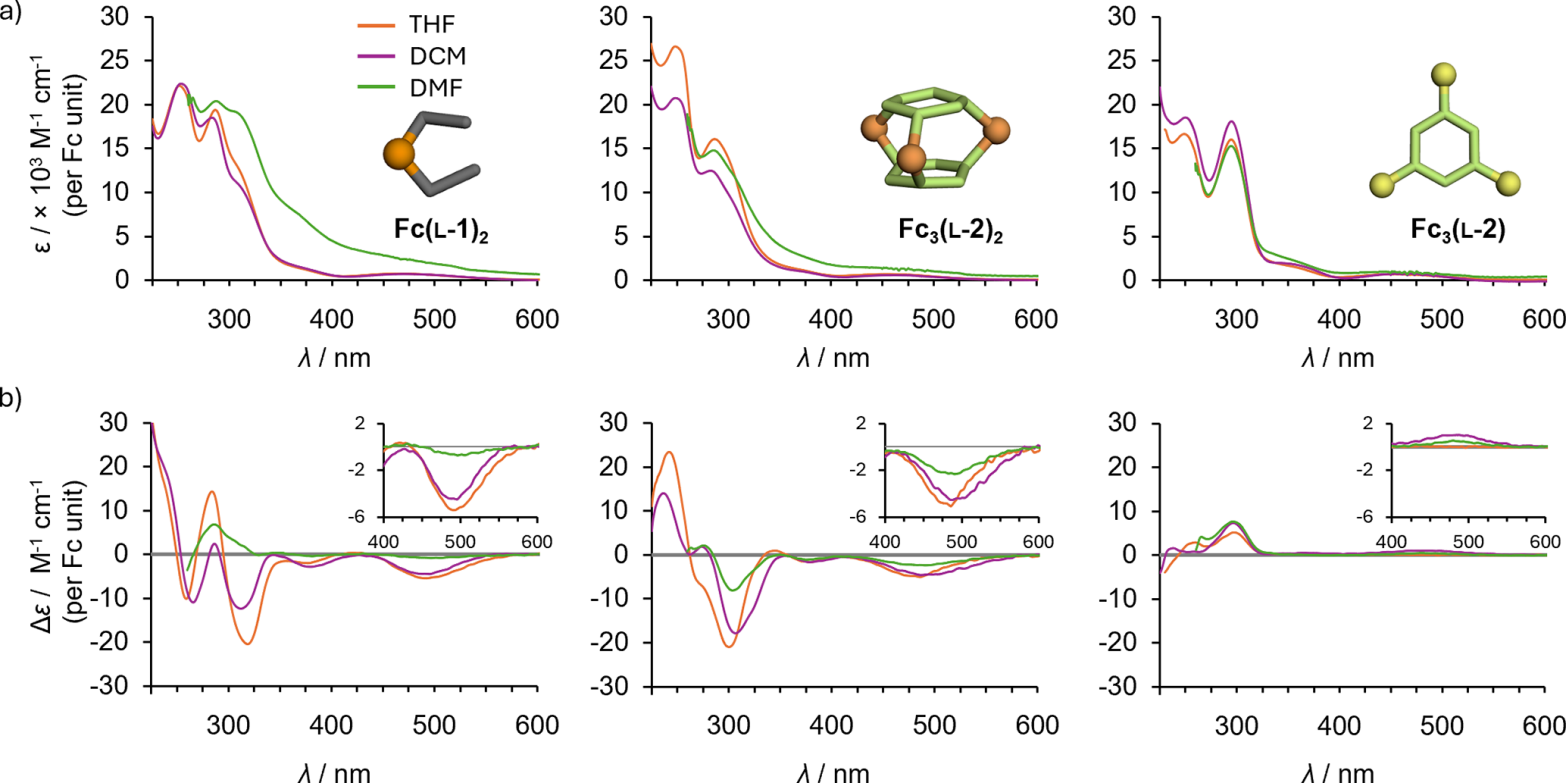
Comparison of (a) UV–vis and (b) CD spectra for **Fc­(** **l** **-1)**_**2**_, **Fc**_**3**_ **(** **l** **-2)**_**2**_, and **Fc**_**3**_ **(** **l** **-2)** in different solvents (THF—orange, DCM—purple, DMF—green). Insets in CD spectra show zooms in the 400–600 nm range.

To gain insight into the structural origin of the observed CD effects, theoretical calculations were performed (geometry optimizations and time-dependent calculations, all at the M06/Def2SVP//ωB97XD/Def2SVP level with SMD solvation model for THF or DCM).^39^ The initial models were constructed for an analog of **Fc­(** **l** **-1)**_**2**_ by replacing l-Phe with l-alanine (l-Ala) (to reduce computational cost), assuming the most stable *E* configuration of the acylhydrazone double bond and retaining *C*_2_ symmetry. Ten starting conformations, differing in the torsion angle at the Fc unit (α = 0°–(+180)° for P configuration, α = 0°–(−180°) for M configuration), in 36° increments, were examined. After geometry optimization, the set of initial conformations converged to 5 structures with similar energies (within a calculation’s error, Δ*G* < 4 kJ mol^–1^, Table S1, Figure S21). Notably, no intramolecular hydrogen bonds stabilizing any of the energy-minimized conformations were identified, suggesting that induction of the chiral Fc conformation is predominantly of steric origin and/or determined by dipole–dipole interactions.

The conformation of **Fc­(** **l** **-1)**_**2**_ was chosen based on two criteria: the best match between calculated and experimental CD spectra, and compatibility with cage formation. In the selected conformation (Conf.3, Δ*G* = +2.2 kJ mol^–1^, Figure 5a), the Fc unit has *P* axial chirality (syn rotamer), and the torsion angle between the Cp rings is about +56°. The acylhydrazone carbonyl groups adopt an antiparallel arrangement, which can be viewed as a stabilizing dipole–dipole interaction. Because dipole–dipole interaction is electrostatic in nature, it is expected to be strongly solvent dependent, which may explain why a highly polar solvent such as DMF reduces the CD intensity in the region of the d–d transitions of the Fc units. The agreement between the CD spectrum calculated for this conformation and the experimental spectrum of **Fc­(** **l** **-1)**_**2**_ is very good (Figure 5b). Analysis of the Natural Transition Orbitals (NTOs) for the seven lowest–energy transitions (500–300 nm) shows that the first six transitions are dominated by Fc-centered orbitals, with only minor contributions from the acylhydrazone linker (for example, the NTO for high-intensity transition #4, Figure 5b; other NTOs are shown in Figure S22). This indicates that, in this region, the sign of the CD bands is determined mainly by the axial chirality of the Fc units. A noticeable contribution from acylhydrazone orbitals is seen only for transition #7 (Figure 5c).

**Figure 5:**
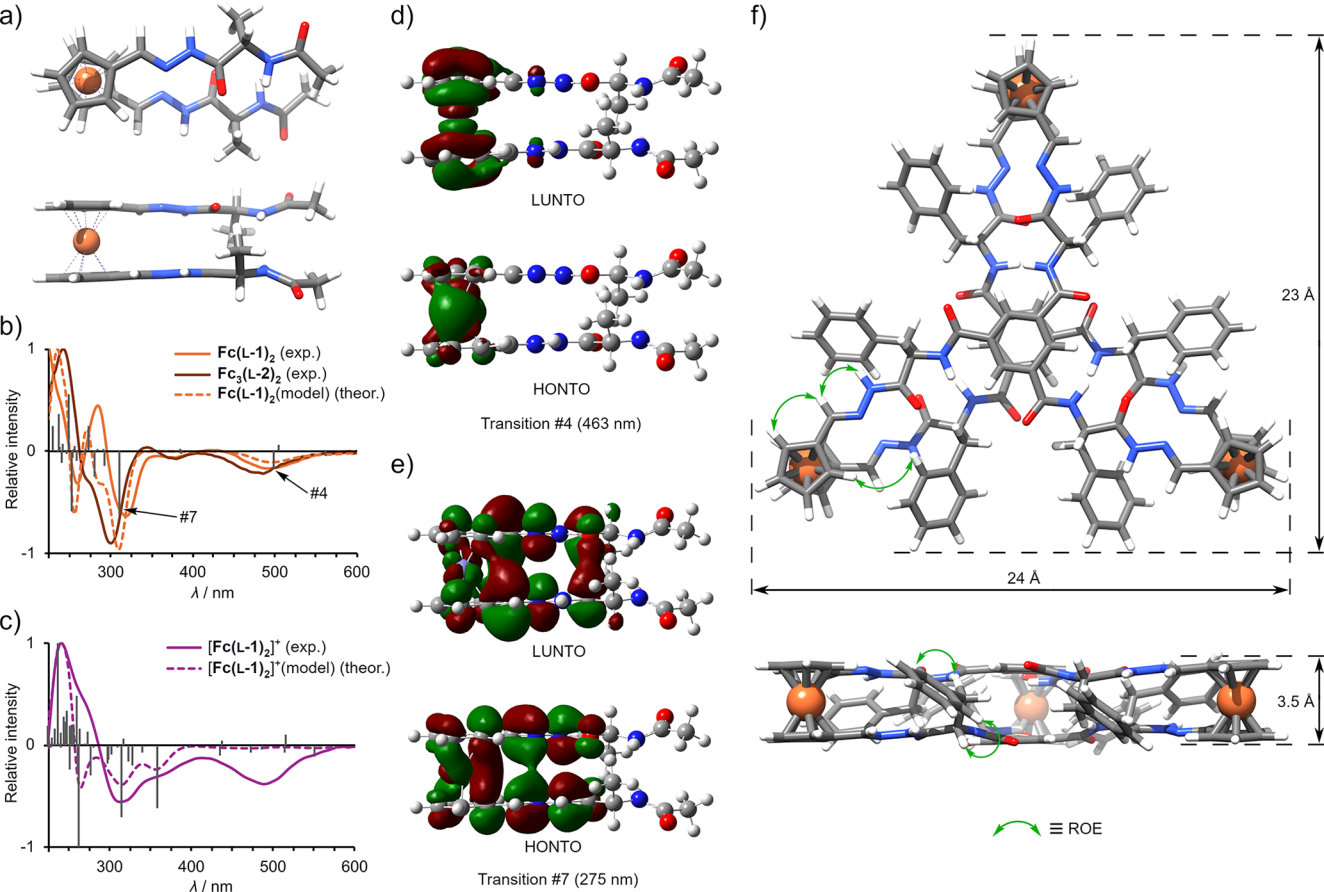
Theoretical calculations: (a) geometry-optimized model of **Fc­(** **l** **-1)**_**2**_ (M06/Def2SVP/SMD­(THF) without Ph rings to reduce computational cost); (b) comparison of experimental and theoretical ECD spectra of **Fc­(** **l** **-1)**_**2**_ and **Fc**_**3**_ **(** **l** **-2)**_**2**_ (M06/Def2SVP/SMD­(THF)//ωB97XD/Def2SVP/SMD­(THF)); (c) comparison of experimental and theoretical ECD spectra of **Fc­(** **l** **-1)**_**2**_ after electrochemical oxidation (M06/Def2SVP/SMD­(DCM)//ωB97XD/Def2SVP/SMD­(DCM); (d) HONTO and LUNTO for transition #4 (80% contribution) of **Fc­(** **l** **-1)**_**2**_; (e) HONTO and LUNTO for transition #7 (76% contribution) of **Fc­(** **l** **-1)**_**2**_; and (f) model of **Fc**_**3**_ **(** **l** **-2)**_**2**_ with marked ROE interactions and structural dimensions.

Based on the optimized geometry of **Fc­(** **l** **-1)**_**2**_, and taking into account analogous CD and NMR spectra, the model of **Fc**_**3**_ **(** **l** **-2)**_**2**_ was constructed (Figure 5f). **Fc**_**3**_ **(** **l** **-2)**_**2**_ model has a double-decker structure (distance between stacking central rings is 3.41 Å) and the intramolecular contacts that are fully consistent with the cross-peaks observed in the ^1^H–^1^H ROESY spectrum, thereby providing strong support for the proposed model. Specifically, the cross peaks between signals of C–H ^e^ and those from Fc units: H^e^(CH) ↔ H^a^(Fc) (δ 7.92 ↔ 4.95 ppm); H^e^(CH) ↔ H^d^(Fc) (δ 7.92 ↔ 4.50 ppm); and between ortho-protons from l-phe: H^o^(Ph-ortho) ↔ H^i^(NH) (δ 7.40 ↔ 8.78 ppm); H^o^(Ph-ortho) ↔ H^a^(Fc) (δ 7.40 ↔ 4.95 ppm); and H^o^(Ph-ortho) ↔ H^g^(α) (δ 7.40 ↔ 6.09 ppm). Those contacts support the well-defined position of the l-Phe side chain and resulting sharp resonances in the ^1^H NMR spectrum. Additionally, the H^f^(NH) ↔ H^e^(CH) cross peak (δ 7.92 ↔ 4.95 ppm) supports the *E* configuration of the acylhydrazone double bond (Figures 2c, 5f, and S9). The abnormally downfield-shifted H^g^ signal can also be explained by this model, as H^g^ lies within the deshielding region of the l-Phe phenyl ring current.

### Electrochemistry

Considering the redox-active Fc units present in these structures, electrochemical characterization was carried out. For **Fc­(** **l** **-1)**_**2**_, a single, fully reversible redox wave (*E*_f_ = 0.44 V vs Ag/Ag^+^, Figure 6a) is observed, which is assigned to the Fc/Fc^+^ redox couple. The cyclic voltammograms (CV) remain unchanged over 10 cycles, indicating the electrochemical stability of the new scaffold. The *E*_f_ value for **Fc­(** **l** **-1)**_**2**_ differs significantly from that of Fc (*E*_f_ = 0.23 V vs Ag/Ag^+^) and Fc­(CHO)_2_ (*E*_f_ = 0.79 V vs Ag/Ag^+^), indicating that this potential is characteristic for the Fc–hydrazone linkage.

**Figure 6:**
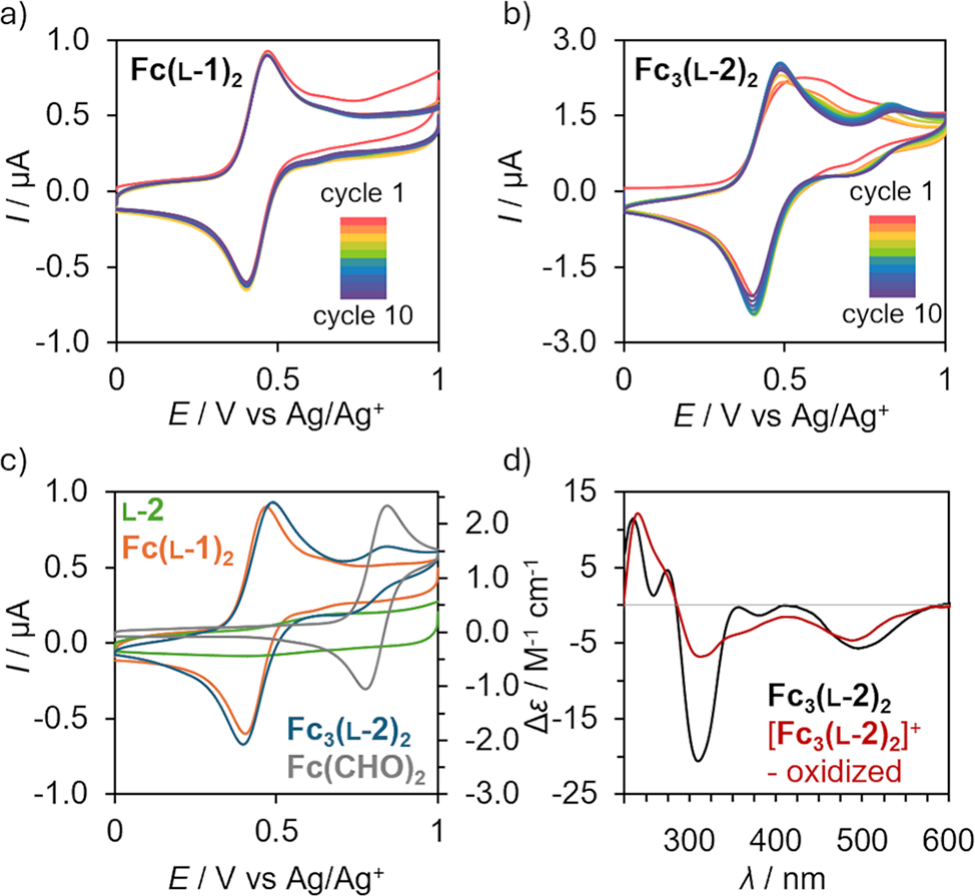
Cyclic voltammograms (CV) for: (a) **Fc­(** **l** **-1)**_**2**_ (10 cycles); (b) **Fc**_**3**_ **(** **l** **-2)**_**2**_ (10 cycles); (c) overlay of CVs of various building blocks (0.1 mM in DCM containing 0.1 M mM Bu_4_NPF_6_ as supporting electrolyte, scan rate 10 mV s^–1^); (d) comparison of CD spectra of **Fc**_**3**_ **(** **l** **-2)**_**2**_ before and after electrochemical oxidation (5 × 10^–5^ M in DCM/0.1 M Bu_4_NPF_6_).

For **Fc**_**3**_ **(** **l** **-2)**_**2**_, a poorly developed oxidation peak is observed in the first CV cycle (Figure 6b). In subsequent cycles, the response evolves, and after approximately the fifth cycle, two well-resolved redox couples are obtained, which then remain unchanged. The first redox process (*E*_f_ = 0.44 V vs Ag/Ag^+^) closely resembles that of **Fc­(** **l** **-1)**_**2**_ and is likewise fully reversible. The peak current shows a linear dependence on the square root of the scan rate for the corresponding reduction peak at approximately 0.40 V, indicating a diffusion-controlled electron-transfer process (see Figure S25). The second redox wave (*E*_f_ = 0.79 V vs Ag/Ag^+^) occurs at a potential similar to the Fc­(CHO)_2_ and exhibits markedly smaller anodic and cathodic peak currents and reduced electrochemical reversibility, as evidenced by the increasing separation of the anodic and cathodic peak potentials with increasing scan rate. The behavior of **Fc**_**3**_ **(** **l** **-2)**_**2**_, in contrast to **Fc­(** **l** **-1)**_**2**_, suggests that structural changes occur during the repeated oxidation–reduction cycles, leading to an equilibrium state that is established after approximately five cycles. Given that the first redox wave of **Fc**_**3**_ **(** **l** **-2)**_**2**_ appears at the same potential as that of **Fc­(** **l** **-1)**_**2**_, whereas the second wave resembles that of Fc­(CHO)_2_, we hypothesize partial electrochemical decomposition, for example, dissociation of Fc­(CHO)_2_ from one of the arms upon oxidation. Such dissociation may facilitate conformational adaptation required to accommodate the three positive charges of [**Fc**_**3**_ **(** **l** **-2)**_**2**_]^3+^ in a low-polarity medium. An alternative explanation is the formation of tight ion pairs between the oxidized ferrocene cage and PF_6_^–^, which can occur in low-polarity solvents such as dichloromethane—appears less likely here, given the diffusion-controlled character of both redox processes and the absence of additional cathodic features on the reverse scan. Finally, the identical *E*_f_ values for **Fc­(** **l** **-1)**_**2**_ and **Fc**_**3**_ **(** **l** **-2)**_**2**_ indicate the absence of significant electronic communication between the Fc units in **Fc**_**3**_ **(** **l** **-2)**_**2**_,^40^ which is readily rationalized by the interruption of conjugation by the sp^3^-hybridized carbons within the amino acid backbone.

Considering the chiral nature of **Fc**_**3**_ **(** **l** **-2)**_**2**_, we were eager to investigate how oxidation of Fc units influences its chiral properties. We employed in situ electrochemical oxidation directly inside the CD spectrometer, using a quartz cuvette dedicated to electrochemical measurements and equipped with a platinum mesh working electrode. The **Fc**_**3**_ **(** **l** **-2)**_**2**_ solution was subjected to a constant current of 0.5 mA, corresponding to 3 equiv. of charge relative to the Fc units present in the sample. The CD spectrum recorded immediately after oxidation revealed subtle changes in the observed bands, namely a decrease in the ∼320 nm band and a slight blue shift of the ∼490 nm band (Figure 6d). The comparison of the experimentally observed changes with the theoretical CD spectrum calculated for a model oxidized structure ([**Fc­(** **l** **-1)**_**2**_]^**+**^, Figure 5c) shows similar trends, suggesting that observed changes may originate from oxidation of **Fc**_**3**_ **(** **l** **-2)**_**2**_, not decomposition.

### Conclusions

In summary, we have introduced a new structural motif, **Fc­(** **l** **-1)**_**2**_, based on a ferrocene unit disubstituted with amino acid derivatives. This motif features (a) intrinsic chirality originating from the amino acid residues, (b) induced axial chirality at the ferrocene core, and (c) a linker incorporating a reversible covalent acylhydrazone bond. **Fc­(** **l** **-1)**_**2**_ maintains its ordered structure in noncompetitive solvents (DCM, THF), exhibiting pronounced circular dichroism responses in the visible range arising from ferrocene-centered electronic transitions. Furthermore, we have demonstrated that this motif can be integrated into multifunctional cores to yield robust *D*_3_-symmetric trigonal platforms, exemplified by **Fc**_**3**_ **(** **l** **-2)**_**2**_. The synthesis of **Fc**_**3**_ **(** **l** **-2)**_**2**_ proceeds with high efficiency (>90%), and due to the reversible nature of the acylhydrazone linkage, narcissistic chiral self-sorting occurs, leading to the selective formation of homochiral platforms **Fc**_**3**_ **(** **l** **-2)**_**2**_ and **Fc**_**3**_ **(** **d** **-2)**_**2**_ from a racemic mixture of precursors.

Given the broad applicability of previously reported ferrocene–amino acid hybrids based on Herrick’s motif, we anticipate comparable or expanded utility for the new **Fc**_**3**_ **(** **l** **-2)**_**2**_ motif. The presence of a dynamic covalent linker, coupled with rigid and highly symmetric chiral building blocks and inherent redox activity, opens pathways to new functional materials. In particular, these features make **Fc**_**3**_ **(** **l** **-2)**_**2**_ not only the first example of highly organized Fc derivative with distinct chirality-related features, but also a promising candidate for the design of chiral redox-active porous architectures, such as molecular cages, and organized materials (e.g., MOFs, COFs), that could harness both chirality and redox properties for advanced applications, including asymmetric electrochemical transformations.

## Experimental Section

### Materials and Methods

All chemicals and solvents were purchased from Fluorochem, Merck, TCI Europe N.V., Carl Roth, Chem-Impex, and Euriso-top, with reagent-grade purity, and were used as received.

NMR spectra were recorded on Bruker 400 MHz, Varian 500 MHz, and Varian 600 MHz instruments and referenced to the residual solvent signal as an internal standard. Coupling constants (*J*) are reported in Hz.

High-resolution ESI and APCI mass spectra were recorded on a MALDI SYNAPT G2-S HDMS spectrometer.

UV-vis and ECD spectra were recorded on a Jasco J-715 spectropolarimeter.

For electrochemical measurements, a three-electrode cell containing a glassy carbon electrode (GCE, ϕ = 3 mm, Mineral, Poland) as the working electrode, Ag/AgNO_3_ (10 mM AgNO_3_ in MeCN) as the reference (BASi, USA), and platinum wire as the counter electrode (MINERAL, Poland) was employed. Prior to each measurement, the GCE was polished to a mirror-like finish using 0.05 μm alumina suspension on a polishing cloth (Buehler). An electrolytic bridge filled with 0.1 M Bu_4_NPF_6_ in DCM separated the reference electrode from the working solution.

All electrochemical measurements were carried out in 0.1 M Bu_4_NPF_6_ in DCM at room temperature under an argon atmosphere using a CHI 1030 potentiostat (CH Instruments, USA). Cyclic voltammetry (CV) measurements were performed at scan rates ranging from 10 to 500 mV/s.

### Calculations

All calculations were performed within the density functional theory (DFT) approach using the Gaussian 16 program suite.^39^ Models were prepared for alanine instead of phenylalanine to save calculation time. Geometry was optimized with the M06 functional, employing the Def2SVP basis set. Solvent effects were considered within the SMD model approach to model the interaction with the solvents (THF or DCM). Vertical excitation energies were determined at the ωB97XD/Def2SVP theory level by means of the time-dependent DFT (TD DFT) approach. The UV-vis absorption spectra were next simulated by overlapping Gaussian functions for each transition, where the width of the band at 1/e height is fixed at 0.2 eV.

### Synthesis

**l** **-1** has been obtained according to the previously reported method, starting from optically pure l-phenylalanine.^33^

^**1**^ **H NMR** (400 MHz, CDCl_3_-*d*): δ 7.35–7.26 (m, 6H), 7.25–7.16 (m, 4H), 7.02 (d, 2H), 6.05 (m, 2H), 4.63–4.53 (m, 2H), 3.80 (s, 4H), 3.13–2.98 (m, 4H), 1.98 (s, 6H).

### Fc­(l-1)_2_

**l** **-1** (45 mg, 0.2 mmol, 2 equiv.) and Fc­(CHO)_2_ (24 mg, 0.1 mmol, 1 equiv.) were dissolved in 5 mL of CHCl_3_ in a 10 mL round-bottom flask. The suspension was then stirred at room temperature for 24 h, during which time the reaction mixture became a clear dark-orange solution. The crude mixture was evaporated to dryness, washed with Et_2_O, and dried under a high vacuum. Yield: 60 mg (93%).

^**1**^ **H NMR** (600 MHz, CDCl3-*d*): δ 10.04 (s, 2H), 7.65 (s, 2H), 7.29–7.11 (m, 12H), 5.69 (m, 2H), 4.97 (s, 2H), 4.552 (s, 2H), 4.549 (s, 2H), 4.36 (s, 2H), 3.11–2.77 (m, 4H), 2.03 (s, 6H). ^**13**^ **C NMR** (150 MHz, CDCl3-*d*) δ 176.74, 173.90, 146.09, 135.96, 129.28, 128.74, 127.29, 79.26, 72.75, 72.68, 70.82, 66.21, 51.06, 37.76, 22.81. **ESI-MS­(−)** calcd for C_34_H_35_FeN_6_O_4_ [M – H]^−^, 647.2069; found, 647.2065.

### l-2/d-2

Chiral trihydrazides **l** **-2** and **d** -**2** have been synthesized according to the previously reported method starting from optically pure l- or d-phenylalanine.^36^

**l** **-2** ^**1**^ **H NMR** (400 MHz, DMSO-*d*_6_): δ 9.35 (s, 3H), 8.78 (d, *J* = 8.5 Hz, 3H), 8.25 (s, 3H), 7.33–7.11 (m, 15H), 4.72 (q, *J* = 8.8 Hz, 3H), 4.27 (s, 6H), 3.10–2.93 (m, 6H).

**d** **-2** ^**1**^ **H NMR** (400 MHz, DMSO-*d*_6_): δ 9.35 (s, 3H), 8.78 (d, *J* = 8.5 Hz, 3H), 8.25 (s, 3H), 7.38–7.12 (m, 15H), 4.72 (q, *J* = 8.8 Hz, 3H), 4.27 (s, 6H), 3.11–2.94 (m, 6H).

### Fc_3_(l-2)_2_

Chiral trihydrazide **l** **-2** (42 mg, 0.06 mmol, 2 equiv.) and Fc­(CHO)_2_ (22 mg, 0.9 mmol, 3 equiv.) were dissolved in 15 mL of SPS grade THF in 25 mL round-bottom flask. The suspension was then stirred at room temperature for 24 h, during which time the reaction mixture became a clear dark-orange solution. The crude mixture was evaporated to dryness, and the resulting solid residue was suspended in MeCN, sonicated, and filtered. The collected solid was washed with MeCN and dried under a high vacuum. Yield: 55 mg (92%).

^**1**^ **H NMR** (600 MHz, THF-*d*_8_): δ 10.80 (s, 6H), 8.79 (d, *J* = 9.8 Hz, 6H), 8.30 (s, 6H), 7.93 (s, 6H), 7.41 (d, *J* = 7.0 Hz, 12H), 7.06 (t, *J* = 7.4 Hz, 12H), 7.00 (t, *J* = 7.4 Hz, 6H), 6.09 (td, *J* = 11.5, 10.7, 3.2 Hz, 6H), 4.96 (s, 4H), 4.66 (s, 4H), 4.51 (s, 4H), 4.39 (s, 4H), 3.09–2.93 (m, 12H). ^**13**^ **C NMR** (150 MHz, THF-*d*_8_): δ 175.95, 164.49, 142.71, 140.68, 134.98, 130.58, 129.07, 128.50, 126.56, 83.66, 71.31, 70.59, 70.32, 68.07, 52.24, 38.46. **ESI-MS­(−)** calcd for C_108_H_95_Fe_3_N_18_O_12_ [M – H]^−^, 2003.5565; found, 2003.5143.

### Fc_3_(d-2)_2_

Isomer **Fc**_**3**_ **(** **d** **-2)**_**2**_ has been obtained in the same way as **Fc**_**3**_ **(** **l** **-2)**_**2**_ using **d** **-2** as starting material. Yield: 52 mg (87%).

^**1**^ **H NMR** (400 MHz, THF-*d*_8_): δ 10.80 (s, 6H), 8.80 (d, *J* = 9.9 Hz, 6H), 8.30 (s, 6H), 7.93 (s, 6H), 7.41 (d, *J* = 7.1 Hz, 12H), 7.03 (dt, *J* = 24.8, 6.6 Hz, 6H), 6.09 (t, *J* = 9.8 Hz, 6H), 4.96 (s, 4H), 4.66 (s, 4H), 4.51 (s, 4H), 4.39 (s, 4H), 3.06–2.96 (m, 12H). **APCI-MS­(−)** calcd for C_108_H_95_Fe_3_N_18_O_12_ [M – H]^−^, 2003.5565; found, 2003.5436.

### Fc_3_(l-2)

Chiral trihydrazide **l** **-2** (42 mg, 0.06 mmol, 1 equiv.) and Fc­(CHO) (52 mg, 0.24 mmol, 4 equiv.) were dissolved in 15 mL of SPS grade THF in a 25 mL round-bottom flask. The suspension was then stirred at room temperature for 24 h, during which time the reaction mixture became a clear dark-orange solution. The crude mixture was evaporated to dryness, and the resulting solid residue was suspended in Et_2_O, sonicated, and filtered. The collected solid was washed with Et_2_O and dried under a high vacuum. Yield: 70 mg (91%).

^**1**^ **H NMR** (400 MHz, THF-*d*_8_): δ 10.90–10.34 (m, 3H), 8.48–7.76 (m, 6H), 7.58–7.04 (m, 18H), 5.81 (s, 3H), 4.66 (dd, *J* = 38.0, 14.8 Hz, 6H), 4.35 (d, *J* = 28.5 Hz, 6H), 4.15 (d, *J* = 14.3 Hz, 15H), 3.45–3.02 (m, 6H). **APCI-MS­(−)** calcd for C_69_H_62_Fe_3_N_9_O_6_ [M – H]^−^, 1280.2871; found, 1280.2870.

## Supplementary Material



## Data Availability

The data supporting the findings of this study (NMR spectra, UV–vis and CD spectra, computational data) are openly available at: 10.18150/IHRVVI.

## Notes

Synthetic procedures, NMR spectra, mass spectra, calculation details, and electrochemical details (PDF)
