# Supplementary material for: Axial Chirality Induction in Ferrocene-Amino Acid Hybrids—toward Chiral Redox-Active Building Blocks
Source: Inorg Chem. 2026 May 4;65(19):10783–91. doi: 10.1021/acs.inorgchem.6c01109 (PMC13360310; doi:10.1021/acs.inorgchem.6c01109)
Supplement: Supplementary file 1 [file ic6c01109_si_001.pdf]

Electronic Supporting Information for

Axial Chirality Induction in Ferrocene-Amino Acid Hybrids - Toward Chiral Redox-Active Building Blocks

Marcin Konopka,[a] Rafał Śliwa,[b] Marek P. Szymański,[a] Aleksandra Tobolska,[b] Wojciech Wróblewski,[b] Ireneusz Tomczyk,[a] Katarzyna Rybicka-Jasińska,[a] Artur Kasprzak,[b*] Agnieszka Szumna[a*]

[a] *Institute of Organic Chemistry, Polish Academy of Sciences, Kasprzaka 44/52, 01-224 Warsaw, Poland*
[b] *Faculty of Chemistry, Warsaw University of Technology, 00-664 Warsaw, Poland*

Corresponding authors:
**Agnieszka Szumna: agnieszka.szumna@icho.edu.pl*
**Artur Kasprzak: artur.kasprzak@pw.edu.pl*

Table of contents

Materials and methods 2
Synthesis 2
Spectroscopic data 6
Calculations 16
Electrochemistry 23

Materials and methods

All chemicals and solvents were purchased from Fluorochem, Merck, TCI Europe N.V., Carl Roth, Chem-Impex, and Euriso-top, with reagent-grade purity, and were used as received.

NMR spectra were recorded on Bruker 400 MHz, Varian 500 MHz, and Varian 600 MHz instruments and referenced to the residual solvent signal as an internal standard. Coupling constants (*J*) are reported in Hz.

High-resolution ESI and APCI mass spectra were recorded on a MALDI SYNAPT G2-S HDMS spectrometer.

UV-vis and ECD spectra were recorded on a Jasco J-715 spectropolarimeter.

Synthesis

L-1

L-1 has been obtained according to the previously reported method, starting from optically pure L-Phenylalanine.[1]

^{1}H NMR (400 MHz, $CDCl_3$-*d*) δ 7.35 – 7.26 (m, 6H), 7.25 – 7.16 (m, 4H), 7.02 (d, 2H), 6.05 (m, 2H), 4.63 – 4.53 (m, 2H), 3.80 (s, 4H), 3.13 – 2.98 (m, 4H), 1.98 (s, 6H).

Fc(L-1)$_2$

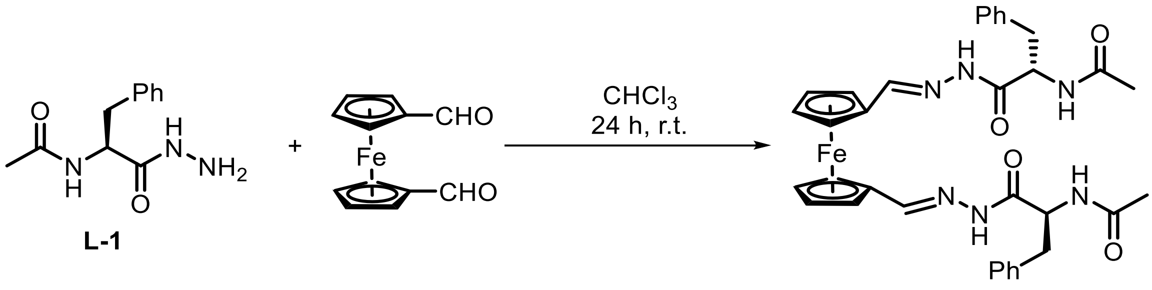

L-1 (45 mg, 0.2 mmol, 2 equiv.) and **Fc(CHO)$_2$** (24 mg, 0.1 mmol, 1 equiv.) were dissolved in 5 mL of $CHCl_3$ in a 10 mL round-bottom flask. The suspension was then stirred at room temperature for 24 h, during which time the reaction mixture became a clear dark-orange solution. The crude mixture was evaporated to dryness, washed with Et_2O, and dried under a high vacuum. Yield: 60 mg (93%).

^{1}H NMR (600 MHz, CDCl3-*d*) δ 10.04 (s, 2H), 7.65 (s, 2H), 7.29 – 7.11 (m, 12H), 5.69 (m, 2H), 4.97 (s, 2H), 4.552 (s, 2H), 4.549 (s, 2H), 4.36 (s, 2H), 3.11 – 2.77 (m, 4H), 2.03 (s, 6H). **^{13}C NMR** (150 MHz, CDCl3-*d*) δ 176.74, 173.90, 146.09, 135.96, 129.28, 128.74, 127.29, 79.26, 72.75, 72.68, 70.82, 66.21, 51.06, 37.76, 22.81. **ESI-MS(-)** calculated for $C_{34}H_{35}FeN_6O_4$ [M-H]$^-$, 647.2069 found 647.2065.

[1] M. Szymański, M. Wierzbicki, M. Gilski, H. Jędrzejewska, M. Sztylko, P. Cmoch, A. Shkurenko, M. Jaskólski, A. Szumna, *Chem. Eur. J.* **2016**, *22*, 3148.

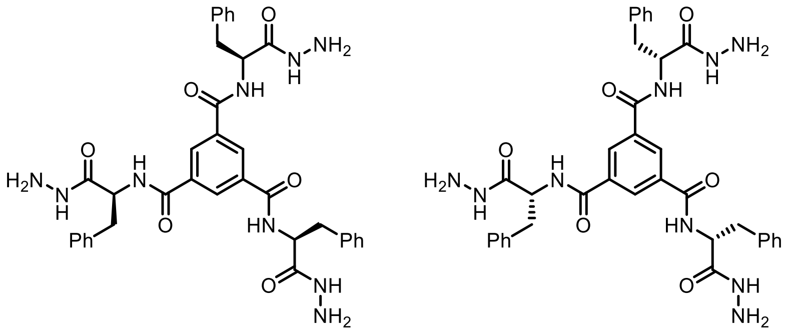

L-2 R-2

L-2 / D-2

Chiral trihydrazides **L-2** and D-**2** have been synthesized according to the previously reported method starting from optically pure L - or D-Phenylalanine.[2]

L-2 ^{1}H NMR (400 MHz, DMSO-*d*$_6$) δ 9.35 (s, 3H), 8.78 (d, J = 8.5 Hz, 3H), 8.25 (s, 3H), 7.33 – 7.11 (m, 15H), 4.72 (q, J = 8.8 Hz, 3H), 4.27 (s, 6H), 3.10 – 2.93 (m, 6H).

D-2 ^{1}H NMR (400 MHz, DMSO-*d*$_6$) δ 9.35 (s, 3H), 8.78 (d, J = 8.5 Hz, 3H), 8.25 (s, 3H), 7.38 – 7.12 (m, 15H), 4.72 (q, J = 8.8 Hz, 3H), 4.27 (s, 6H), 3.11 – 2.94 (m, 6H).

Fc$_3$(L-2)$_2$

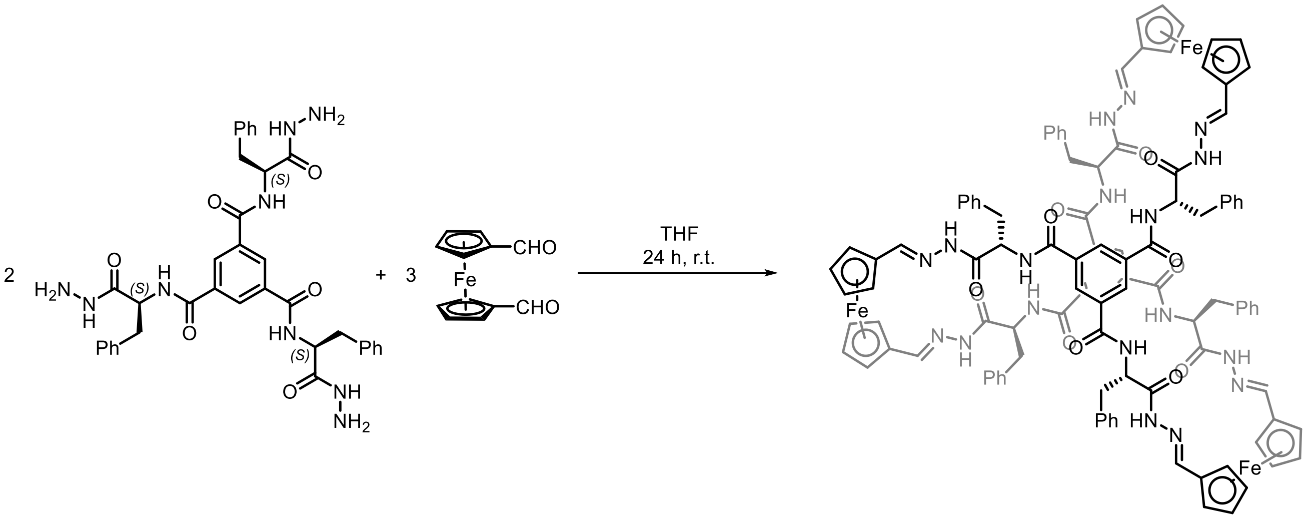

Chiral trihydrazide **L-2** (42 mg, 0.06 mmol, 2 equiv.) and **Fc(CHO)$_2$** (22 mg, 0.9 mmol, 3 equiv.) were dissolved in 15 mL of SPS grade THF in 25 mL round bottom flask. The suspension was then stirred at room temperature for 24 h, during which time the reaction mixture became a clear dark-orange solution. The crude mixture was evaporated to dryness, and the resulting solid residue was suspended in MeCN, sonicated, and filtered. The collected solid was washed with MeCN and dried under a high vacuum. Yield: 55 mg (92%).

[2] M. Grajda, G. Staros, H. Jędrzejewska, A. Szumna, *Inorg. Chem.*, **2022**, 61, 11410-11418.

¹H NMR (600 MHz, THF-d_8) δ 10.80 (s, 6H), 8.79 (d, J = 9.8 Hz, 6H), 8.30 (s, 6H), 7.93 (s, 6H), 7.41 (d, J = 7.0 Hz, 12H), 7.06 (t, J = 7.4 Hz, 12H), 7.00 (t, J = 7.4 Hz, 6H), 6.09 (td, J = 11.5, 10.7, 3.2 Hz, 6H), 4.96 (s, 4H), 4.66 (s, 4H), 4.51 (s, 4H), 4.39 (s, 4H), 3.09 – 2.93 (m, 12H). **¹³C NMR** (150 MHz, THF-d_8) δ 175.95, 164.49, 142.71, 140.68, 134.98, 130.58, 129.07, 128.50, 126.56, 83.66, 71.31, 70.59, 70.32, 68.07, 52.24, 38.46. **ESI-MS(-)** calculated for $C_{108}H_{95}Fe_3N_{18}O_{12}$ $[M-H]^-$, 2003.5565; found 2003.5143.

Fc₃(D-2)₂

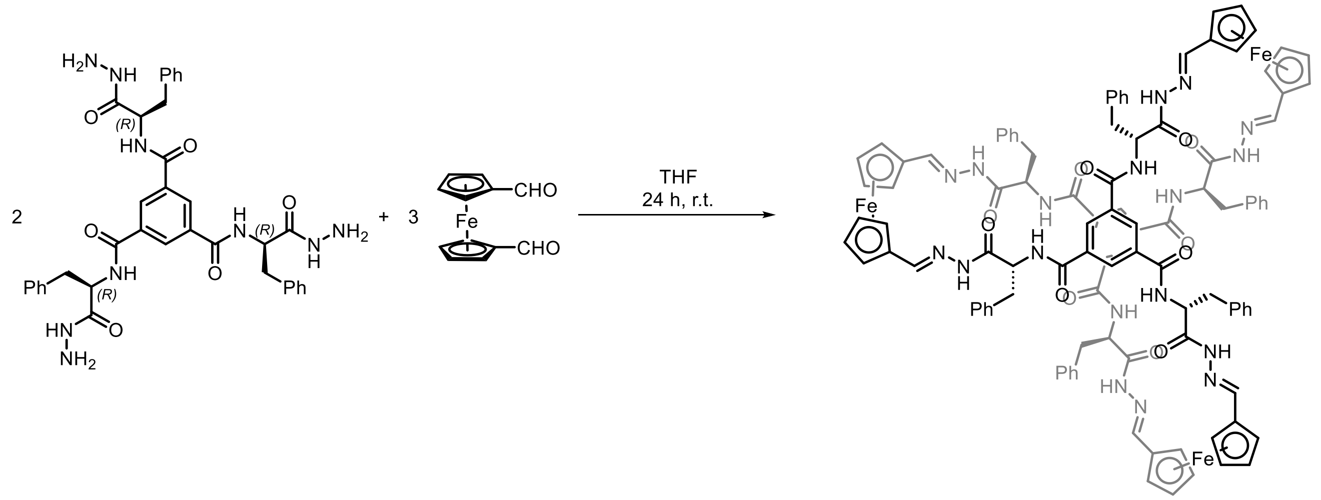

Isomer **Fc₃(D-2)₂** has been obtained in the same way as **Fc₃(L-2)₂** using **D-2** as starting material. Yield: 52 mg (87%).

¹H NMR (400 MHz, THF-d_8) δ 10.80 (s, 6H), 8.80 (d, *J* = 9.9 Hz, 6H), 8.30 (s, 6H), 7.93 (s, 6H), 7.41 (d, *J* = 7.1 Hz, 12H), 7.03 (dt, *J* = 24.8, 6.6 Hz, 6H), 6.09 (t, *J* = 9.8 Hz, 6H), 4.96 (s, 4H), 4.66 (s, 4H), 4.51 (s, 4H), 4.39 (s, 4H), 3.06 – 2.96 (m, 12H). **APCI-MS(-)** calculated for $C_{108}H_{95}Fe_3N_{18}O_{12}$ $[M-H]^-$, 2003.5565; found 2003.5436.

Fc₃(L-2)

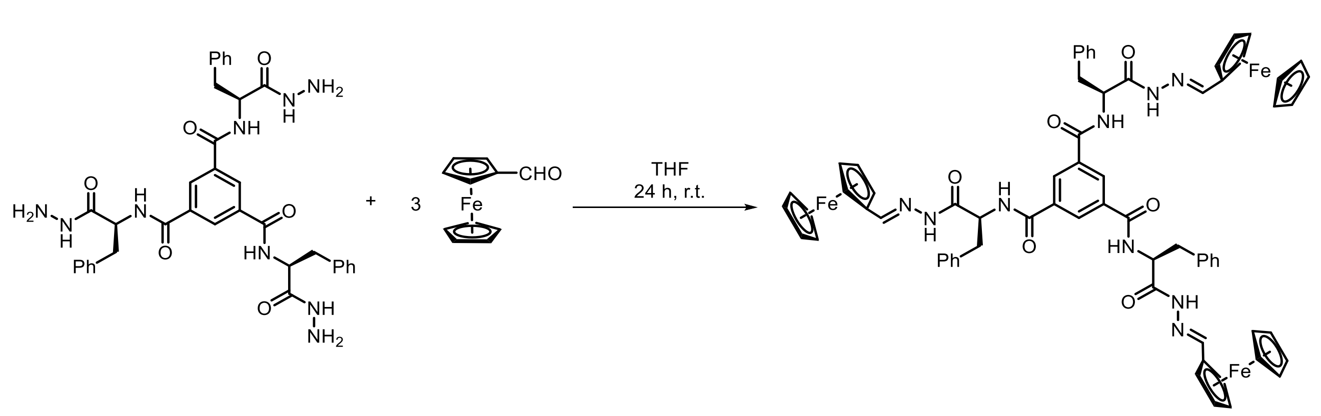

Chiral trihydrazide **L-2** (42 mg, 0.06 mmol, 1 equiv.) and **Fc(CHO)** (52 mg, 0.24 mmol, 4 equiv.) were dissolved in 15 mL of SPS grade THF in a 25 mL round bottom flask. The suspension was then stirred at room temperature for 24 h, during which time the reaction mixture became a clear dark-orange

solution. The crude mixture was evaporated to dryness, and the resulting solid residue was suspended in Et_2O, sonicated, and filtered. The collected solid was washed with Et_2O and dried under a high vacuum. Yield: 70 mg (91%).

^{1}H NMR (400 MHz, THF-d_8) δ 10.90 – 10.34 (m, 3H), 8.48 – 7.76 (m, 6H), 7.58 – 7.04 (m, 18H), 5.81 (s, 3H), 4.66 (dd, J = 38.0, 14.8 Hz, 6H), 4.35 (d, J = 28.5 Hz, 6H), 4.15 (d, J = 14.3 Hz, 15H), 3.45 – 3.02 (m, 6H). **APCI-MS(-)** calculated for $C_{69}H_{62}Fe_3N_9O_6$ $[M-H]^-$, 1280.2871; found 1280.2870.

Spectroscopic data

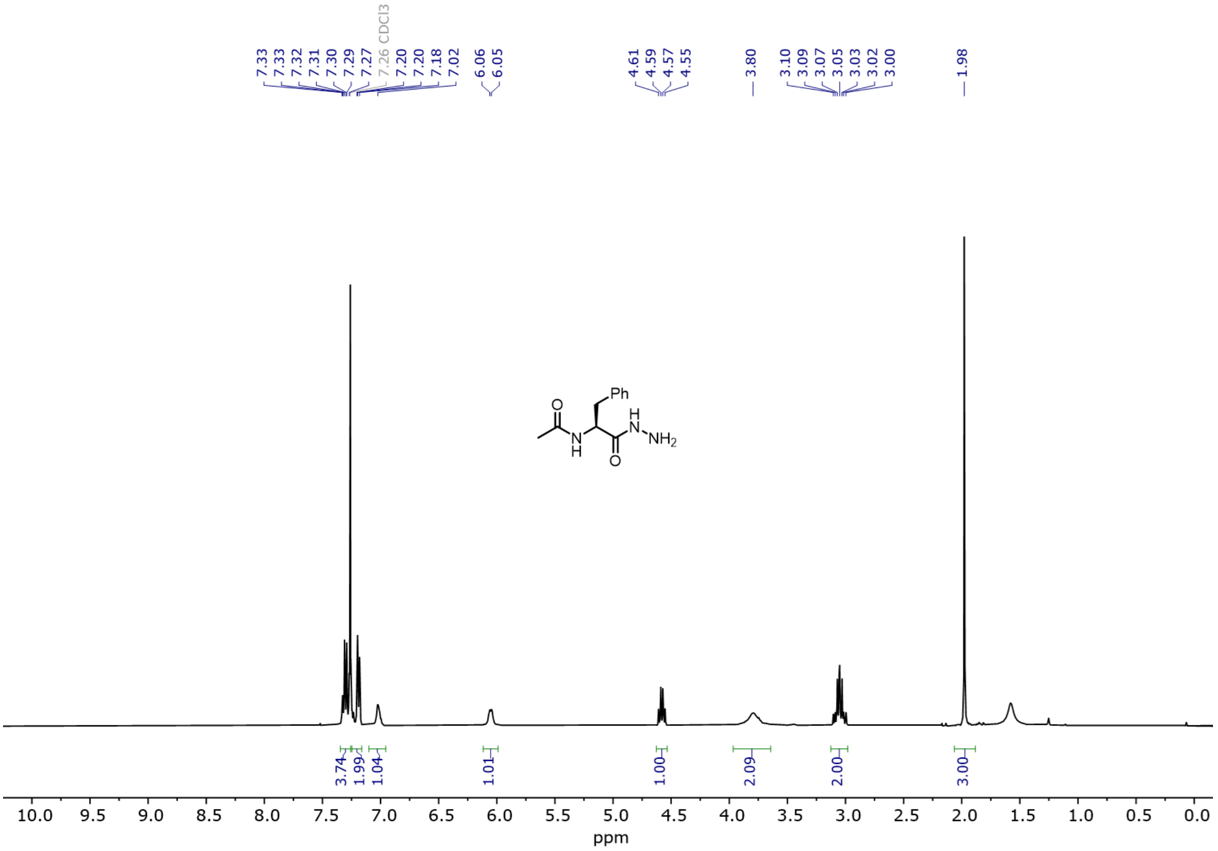

Figure S1. ^{1}H NMR spectrum of **L-1** ($CDCl_3$-*d*, 400 MHz, 298 K).

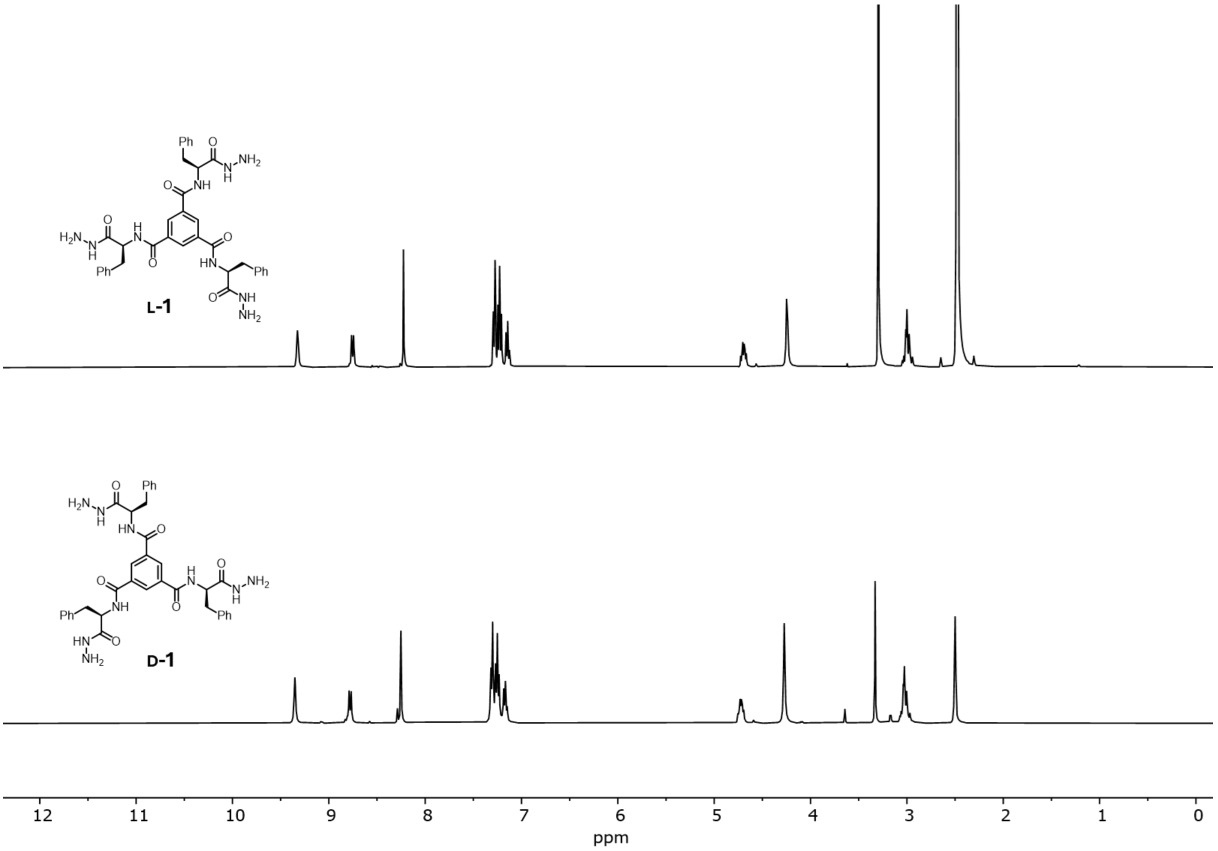

Figure S2. ^{1}H NMR spectra of **L-2** and **D-2** (DMSO-d_6, 400 MHz, 298 K).

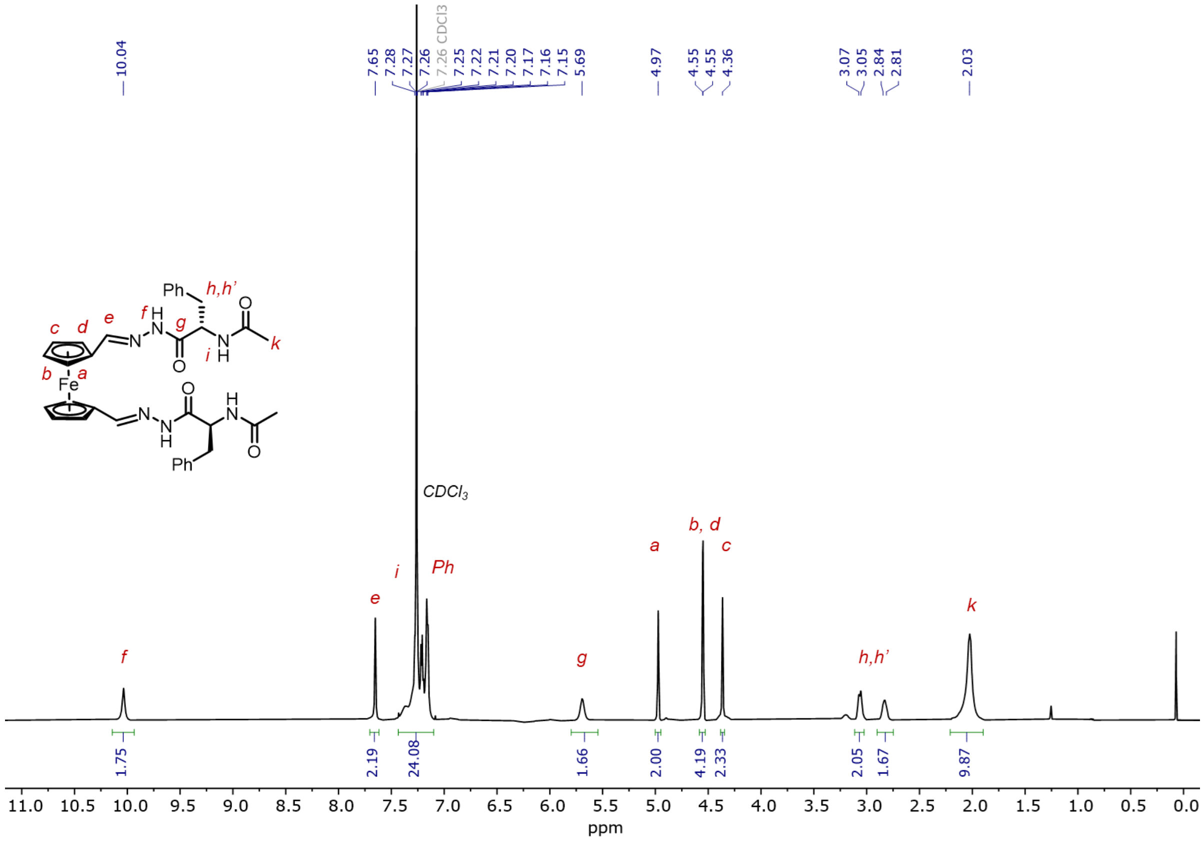

Figure S3. ^{1}H NMR spectrum of **Fc(L-1)$_2$** (THF-d_8, 600 MHz, 298 K).

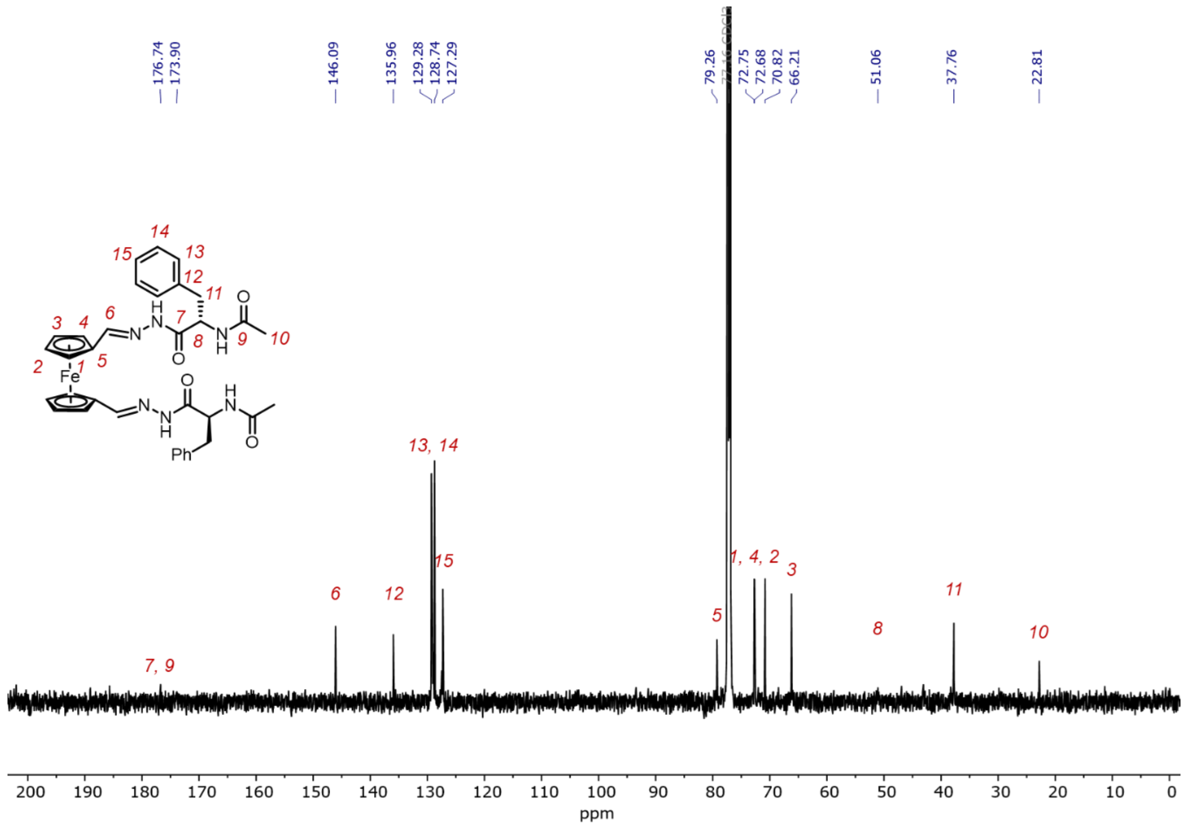

Figure S4. ^{13}C NMR spectrum of **Fc(L-1)$_2$** (THF-d_8, 600 MHz, 298 K).

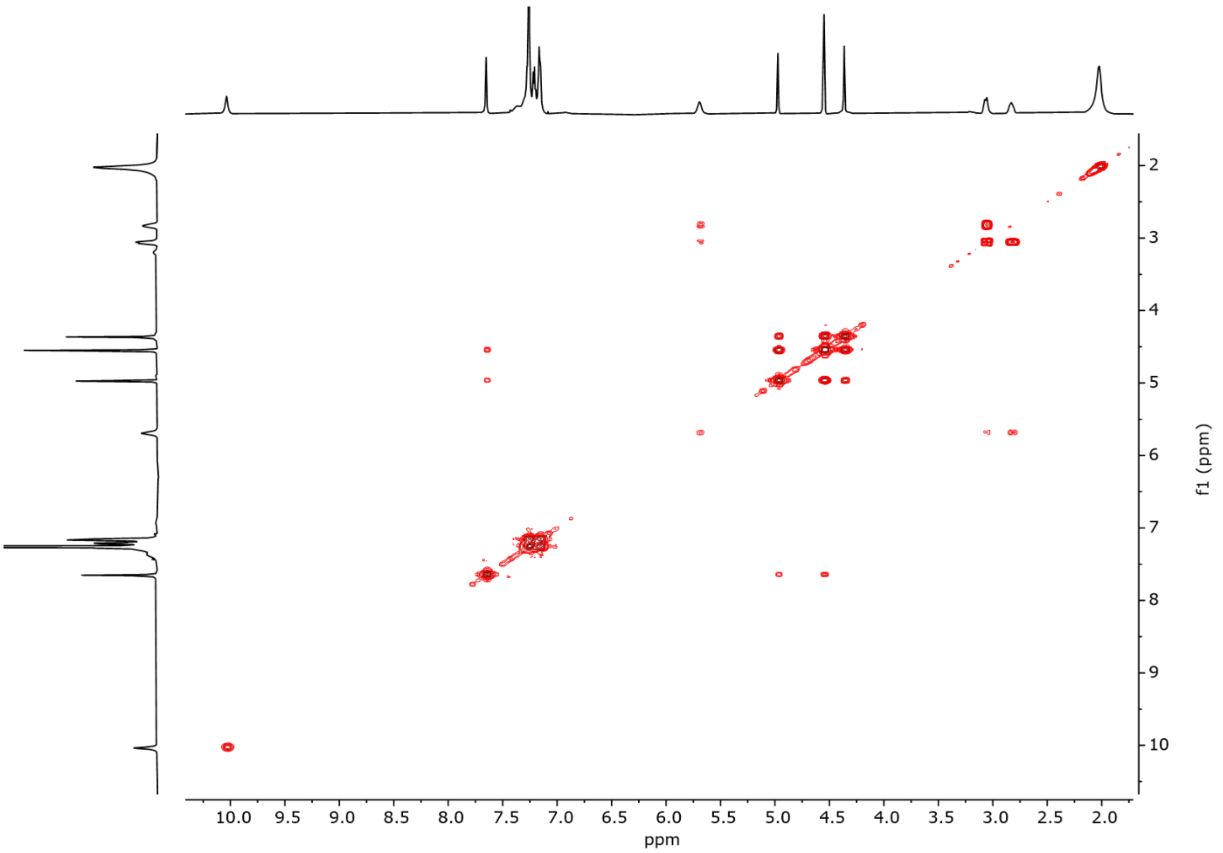

Figure S5. ^{1}H-^{1}H COSY NMR spectrum of **Fc(L-1)$_2$** (THF-d_8, 600 MHz, 298 K).

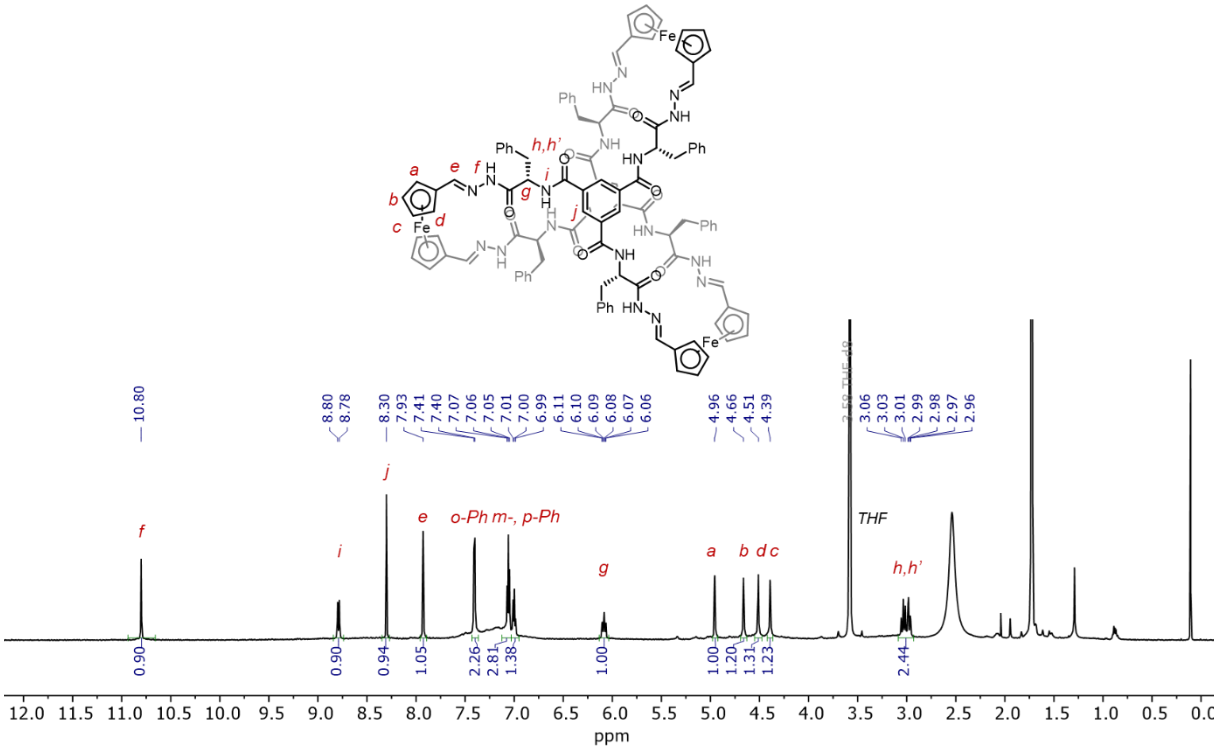

Figure S6. ^{1}H NMR spectrum of **Fc$_3$(L-2)$_2$** (THF-d_8, 600 MHz, 298 K).

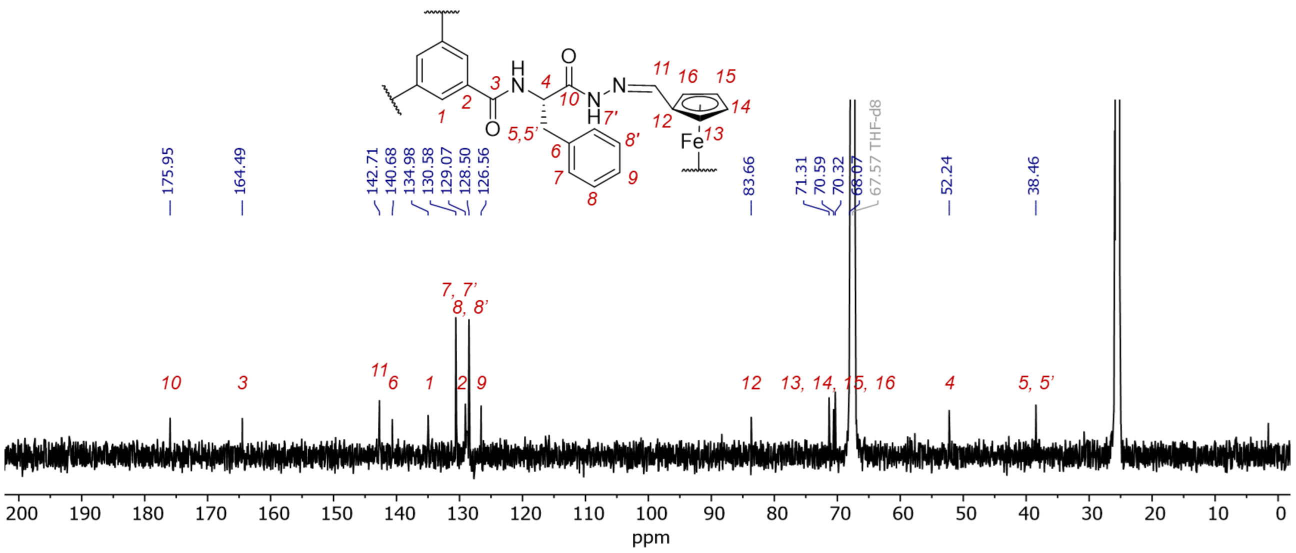

Figure S7. ^{13}C NMR spectrum of **Fc$_3$(L-2)$_2$** (THF-d_8, 150 MHz, 298 K).

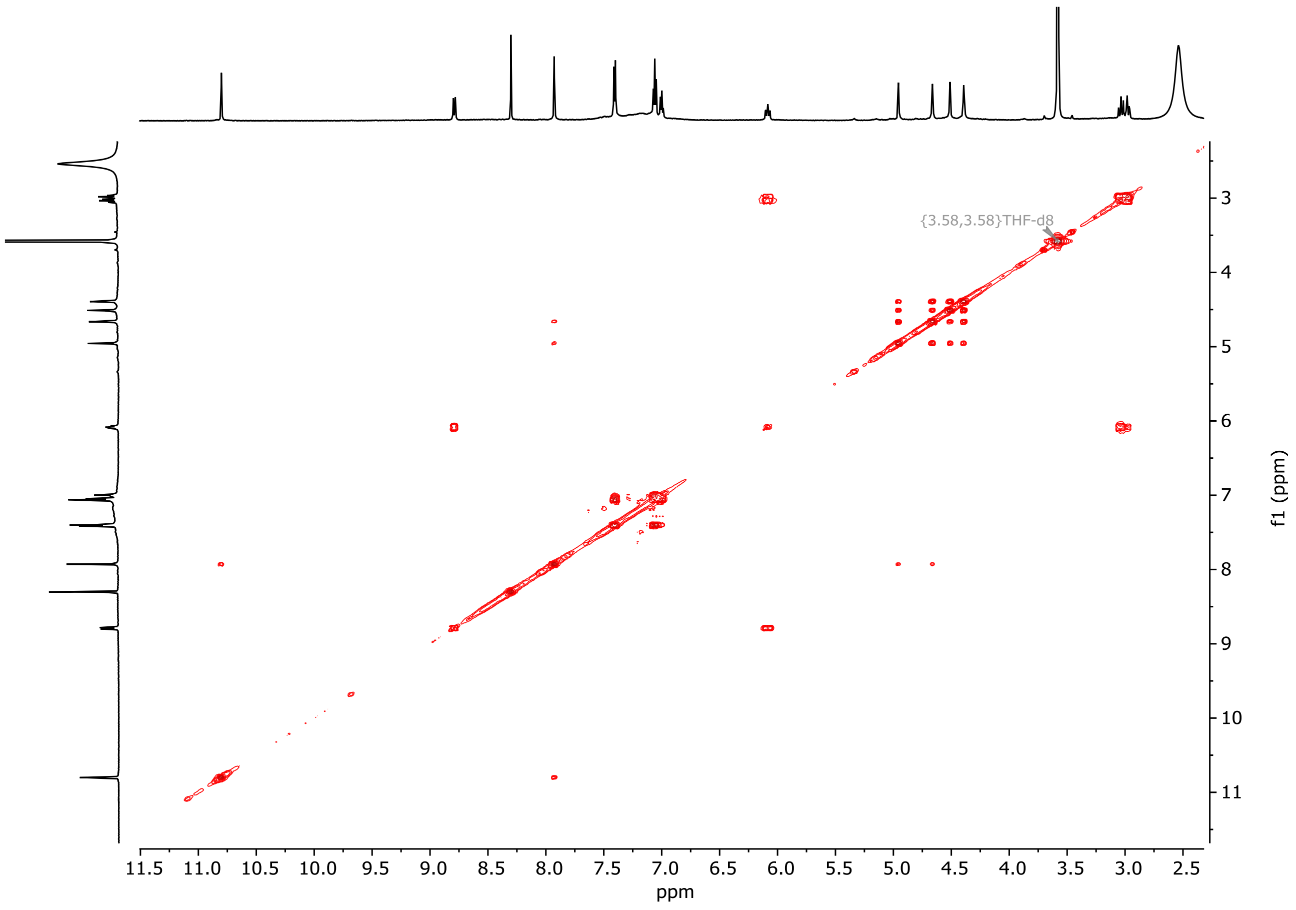

Figure S8. ^{1}H-^{1}H COSY NMR spectrum of **Fc$_3$(L-2)$_2$** (THF-d_8, 600 MHz, 298 K).

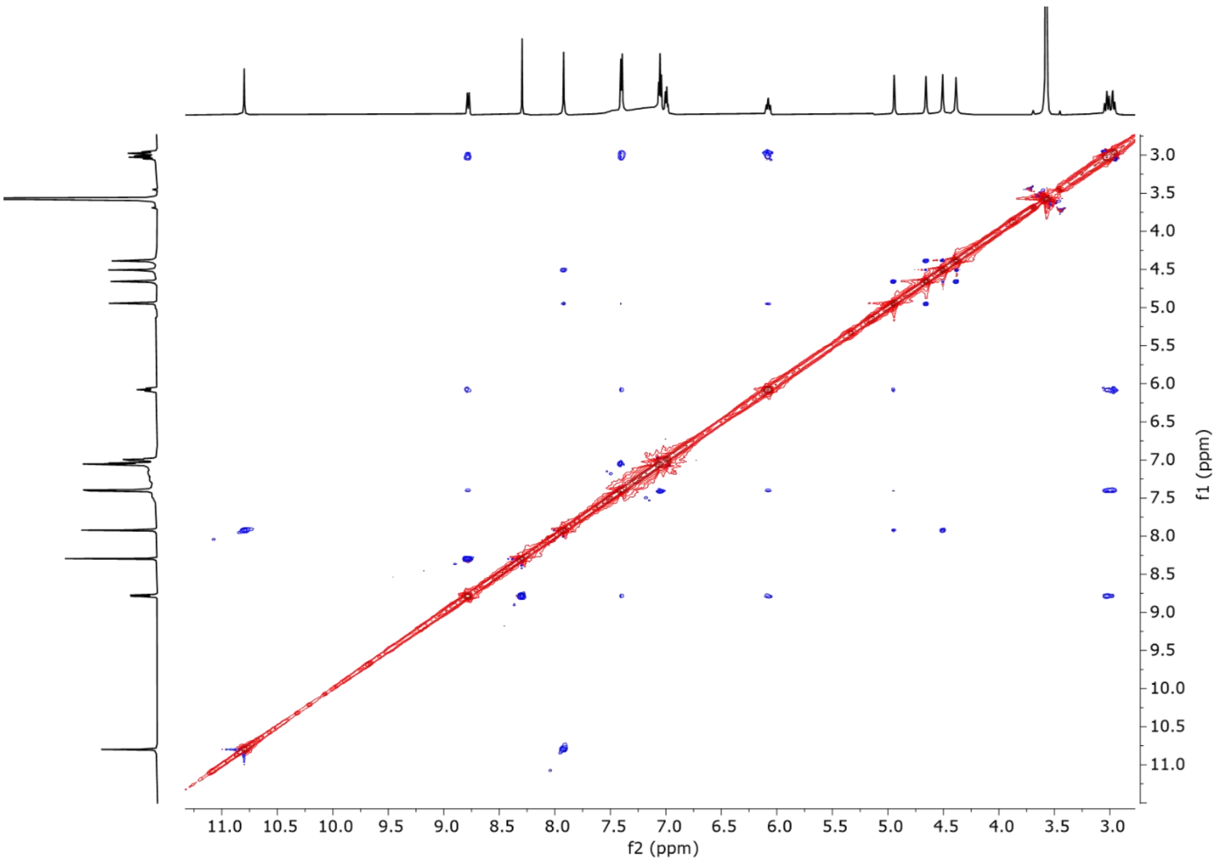

Figure S9. ^{1}H-^{1}H ROESY NMR spectrum of **Fc_3(L-2)$_2$** (THF-d_8, 600 MHz, 298 K).

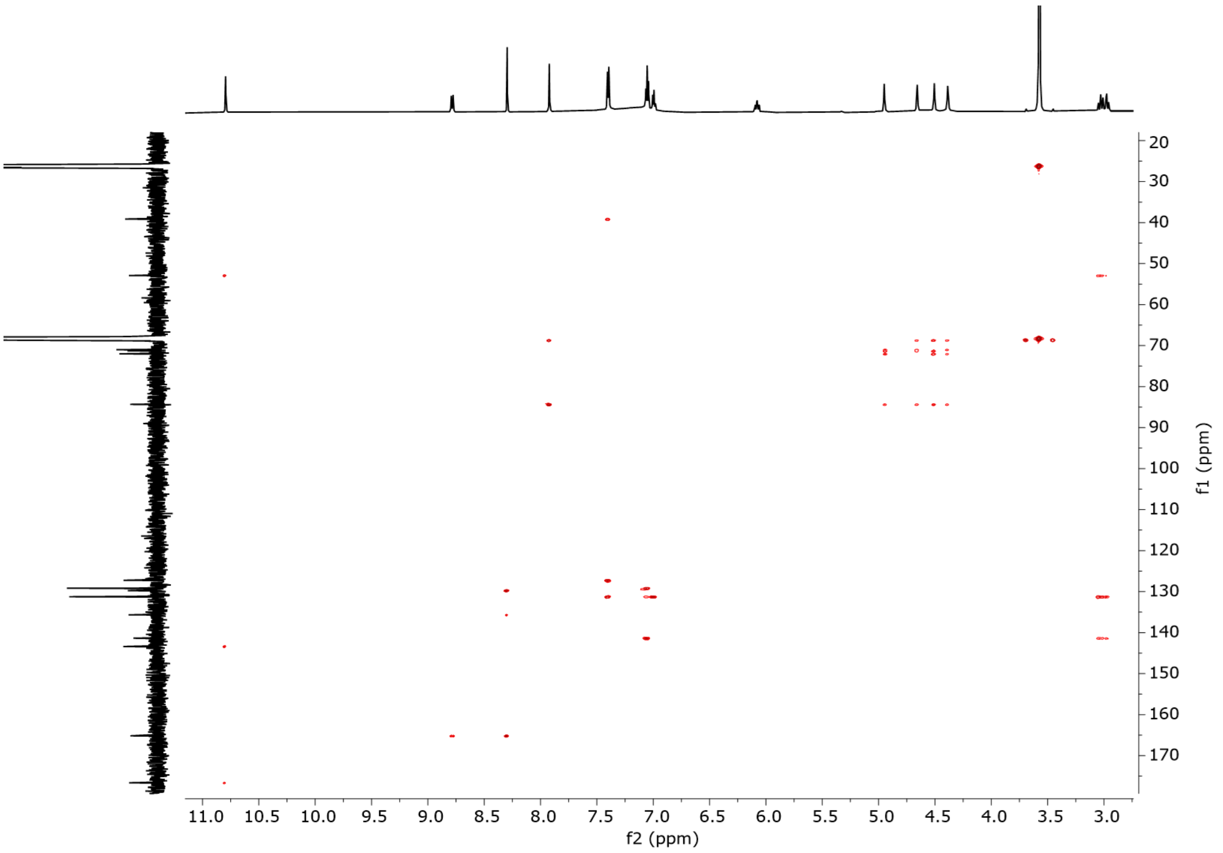

Figure S10. ^{13}C-^{1}H HMBC NMR spectrum of **Fc_3(L-2)$_2$** (THF-d_8, 600 MHz, 298 K).

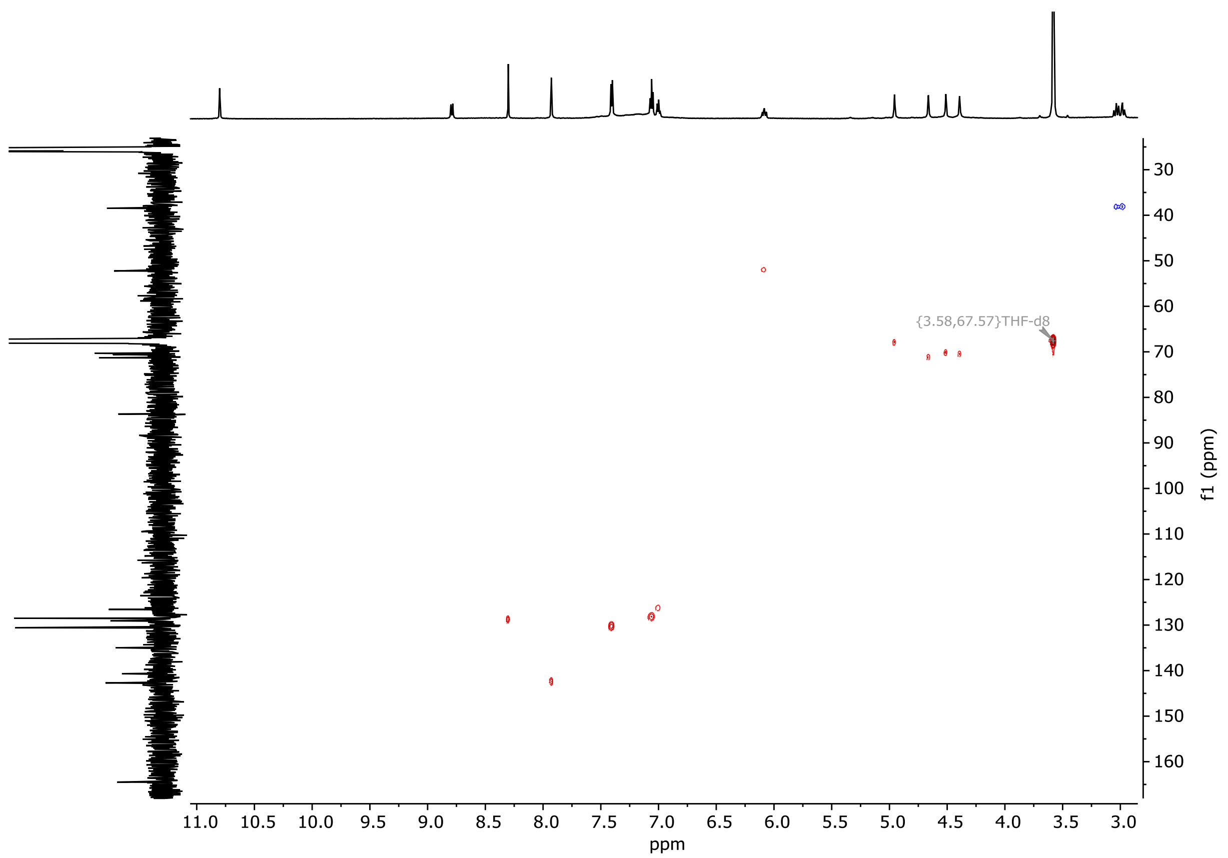

Figure S11. ^{13}C-^{1}H HSQC NMR spectrum of **Fc3(L-2)2** (THF-d_8, 600 MHz, 298 K).

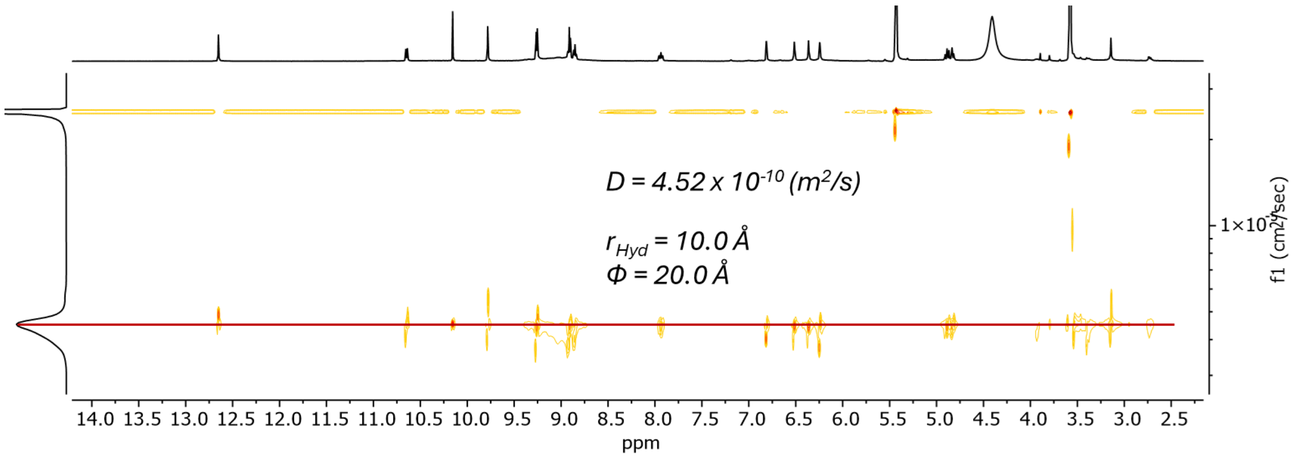

Figure S12. DOSY NMR spectrum of **Fc3(L-2)2** (THF-d_8, 600 MHz, 298 K).

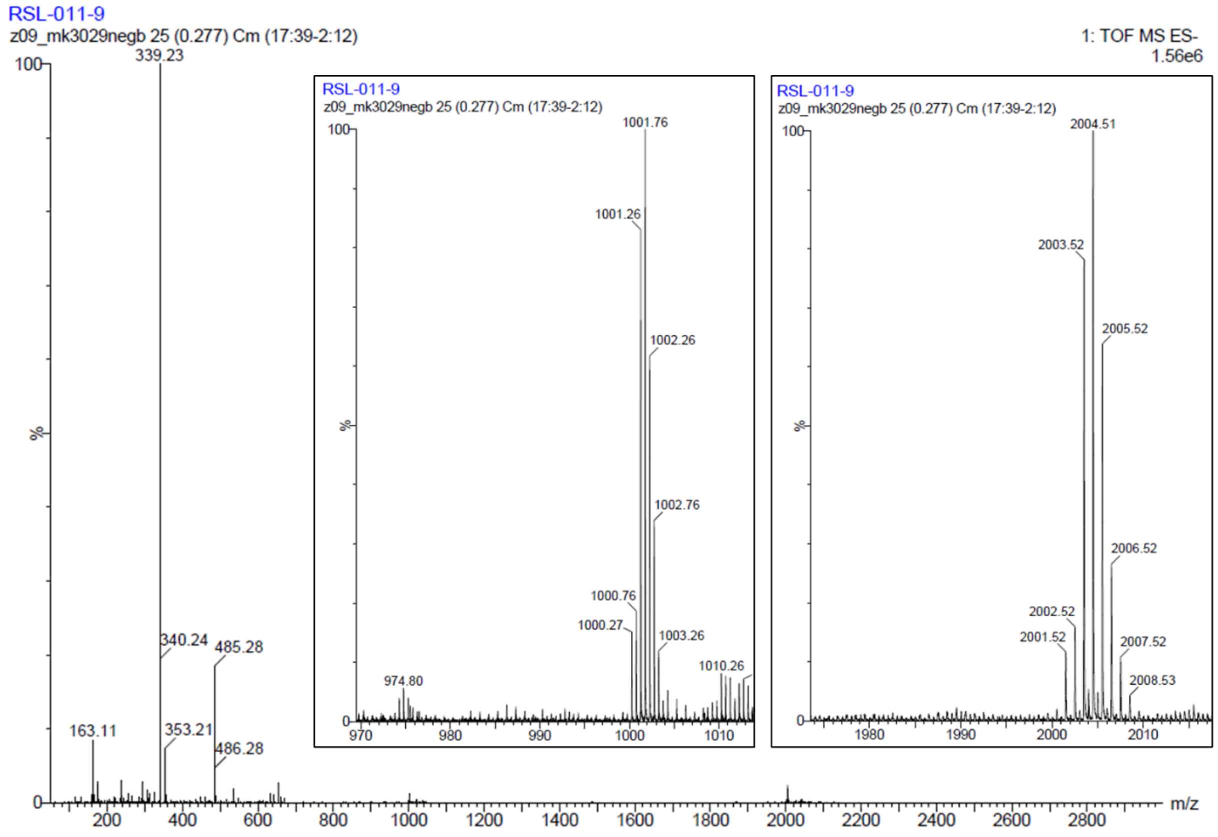

Figure S13. ESI-MS(-) spectrum of **Fc$_3$(L-2)$_2$**.

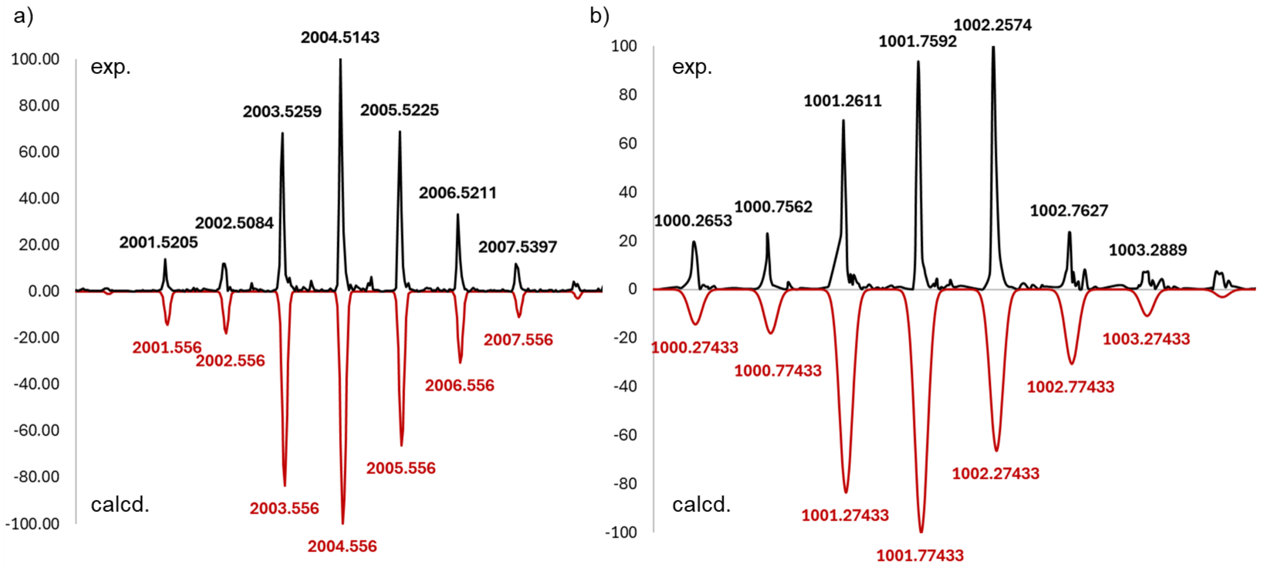

Figure S14. Comparison of **Fc$_3$(L-2)$_2$** spectra, ESI-MS(-) and calculated isotopic patterns: a) calcd. for $C_{108}H_{95}Fe_3N_{18}O_{12}$ (M-H)$^-$, 2004.5560; found 2004.5143; and b) calcd. for $C_{108}H_{95}Fe_3N_{18}O_{12}$ (M-2H)$^-$, 1001.7743; found 1001.7592.

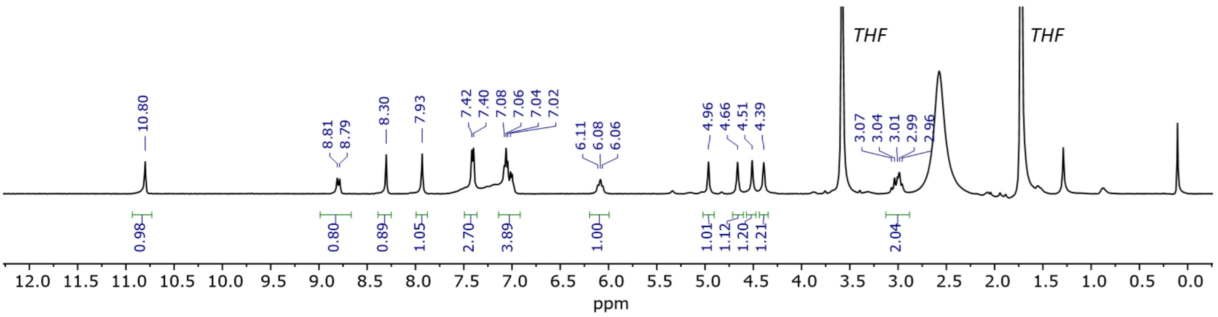

Figure S15. ^{1}H NMR spectrum of **Fc$_3$(D-2)$_2$** (THF-d_8, 400 MHz, 298 K).

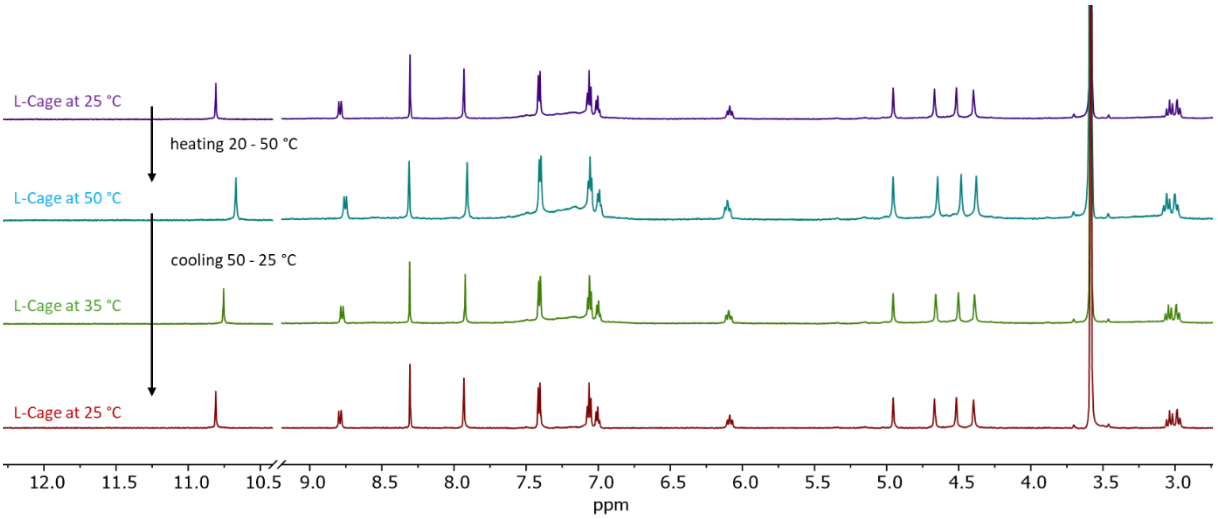

Figure S16. Thermal stability of **Fc$_3$(L-2)$_2$** in 25-50 °C range. Comparison of ^{1}H NMR spectra of **Fc$_3$(L-2)$_2$** at VT (THF-d_8, 600 MHz, 298 - 323 K).

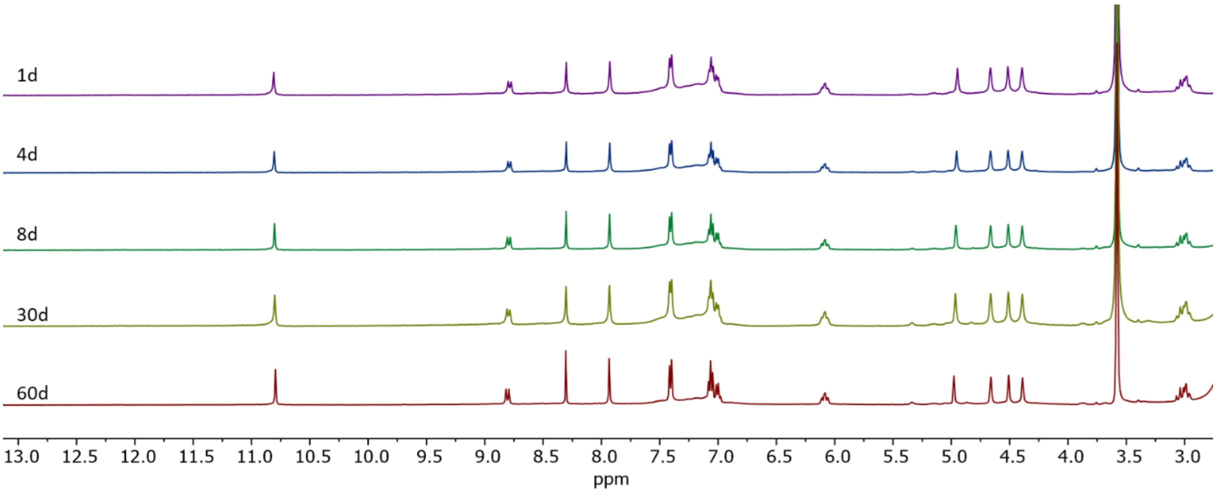

Figure S17. Stability of **Fc$_3$(L-2)$_2$** (range 1 – 60 days). Comparison of ^{1}H NMR spectra of **Fc$_3$(L-2)$_2$** measured from the same sample over time (THF-d_8, 400 MHz, 298 K).

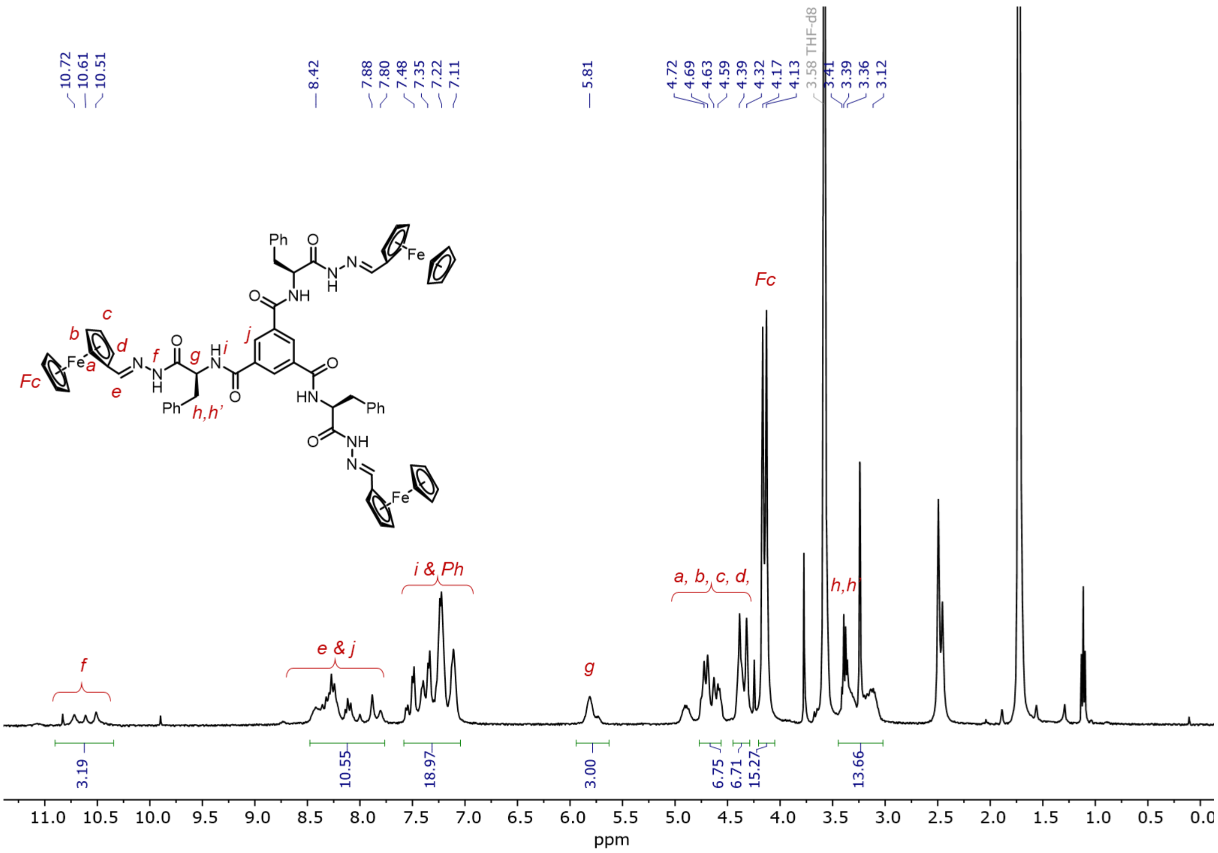

Figure S18. ^{1}H NMR spectrum of **Fc₃(L-2)** (THF-d_8, 600 MHz, 298 K).

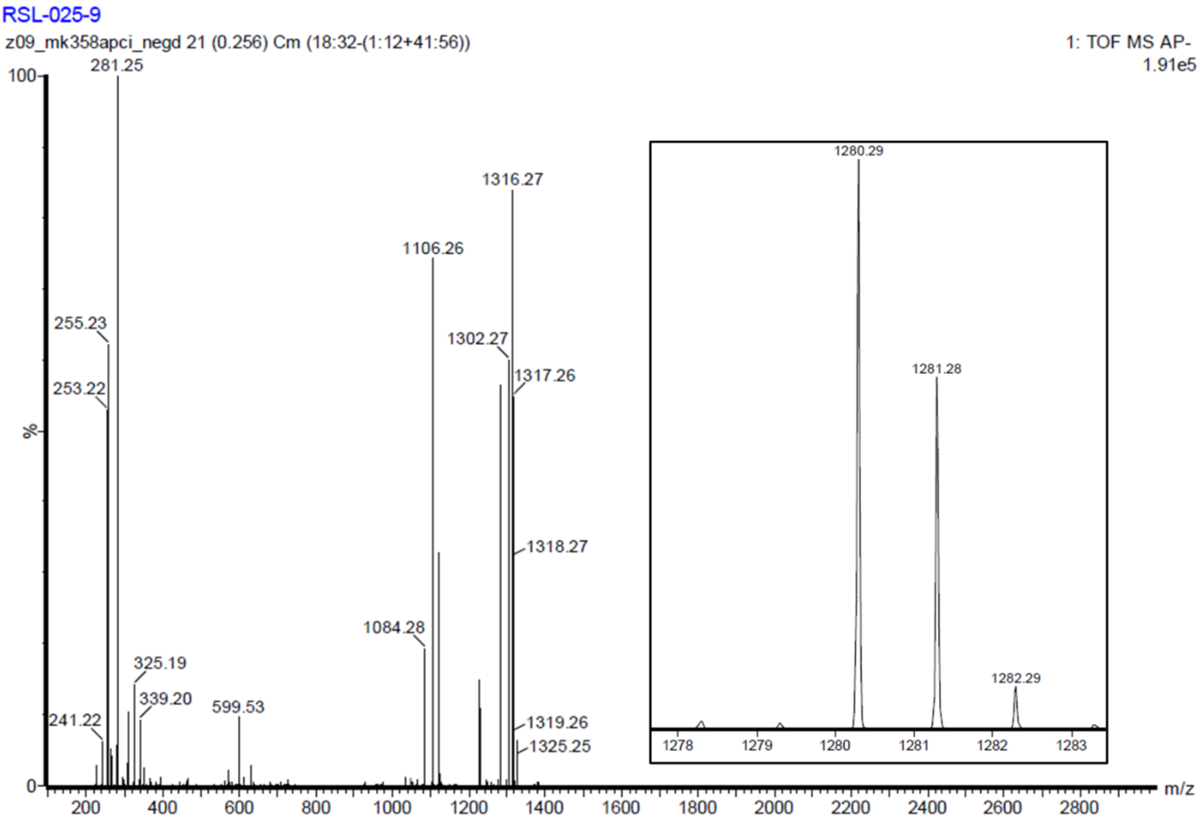

Figure S19. APCI-MS(-) spectrum of **Fc₃(L-2)**.

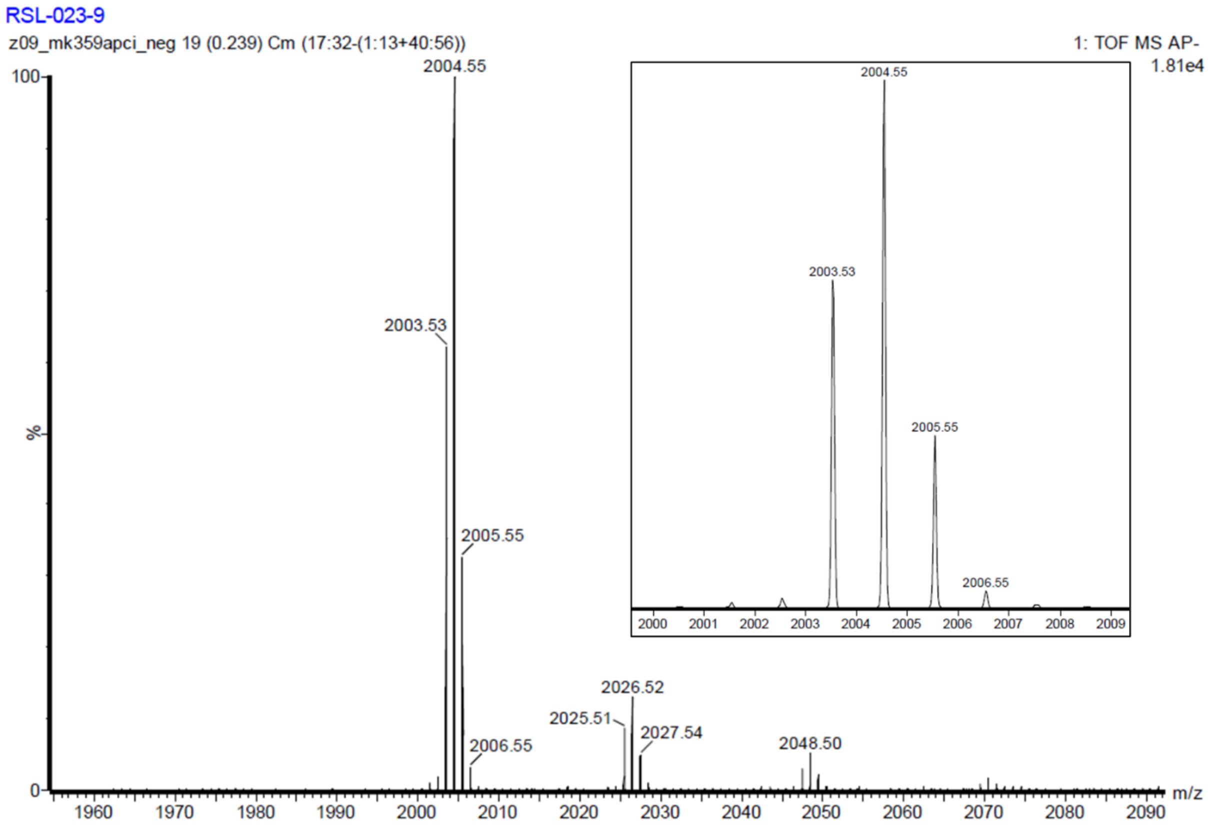

Figure S20. APCI-MS(-) spectrum of **Fc$_3$(D-2)$_2$**.

Calculations

All calculations were performed within the density functional theory (DFT) approach using the Gaussian 16 program suite.[3] Models were prepared for alanine instead of phenylalanine to save calculation time. Geometry was optimized with the M06 functional, employing the Def2SVP basis set. Solvent effects were considered within the SMD model approach to model the interaction with the solvents (THF or DCM). Vertical excitation energies were determined at the ωB97XD/Def2SVP theory level by means of the time-dependent DFT (TD DFT) approach. The UV absorption spectra were next simulated by overlapping Gaussian functions for each transition, where the width of the band at 1/e height is fixed at 0.2 eV.

Table S1. Conformational search for **Fc(L-1)$_2$** (model).

Starting α angle / °	Optimized α angle / °	ΔG / kJ mol^{-1}	Name
-180	-171	0.8	**Conf.2**
-144	-171	0.8	
-108	-100	3.6	**Conf.5**
-72	-22	2.7	
-36	-22	2.7	**Conf.4**
0	-22	6.0	
36	+56	2.2	**Conf.3**
72	+57	3.3	
108	+112	0	**Conf.1**
144	+112	0.1	

[3] M. J. Frisch, G. W. Trucks, H. B. Schlegel, G. E. Scuseria, M. A. Robb, J. R. Cheeseman, G. Scalmani, V. Barone, G. A. Petersson, H. Nakatsuji, X. Li, M. Caricato, A. V. Marenich, J. Bloino, B. G. Janesko, R. Gomperts, B. Mennucci, H. P. Hratchian, J. V. Ortiz, A. F. Izmaylov, J. L. Sonnenberg, D. Williams-Young, F. Ding, F. Lipparini, F. Egidi, J. Goings, B. Peng, A. Petrone, T. Henderson, D. Ranasinghe, V. G. Zakrzewski, J. Gao, N. Rega, G. Zheng, W. Liang, M. Hada, M. Ehara, K. Toyota, R. Fukuda, J. Hasegawa, M. Ishida, T. Nakajima, Y. Honda, O. Kitao, H. Nakai, T. Vreven, K. Throssell, J. A. Montgomery, Jr., J. E. Peralta, F. Ogliaro, M. J. Bearpark, J. J. Heyd, E. N. Brothers, K. N. Kudin, V. N. Staroverov, T. A. Keith, R. Kobayashi, J. Normand, K. Raghavachari, A. P. Rendell, J. C. Burant, S. S. Iyengar, J. Tomasi, M. Cossi, J. M. Millam, M. Klene, C. Adamo, R. Cammi, J. W. Ochterski, R. L. Martin, K. Morokuma, O. Farkas, J. B. Foresman, and D. J. Fox, GAUSSIAN 16 (Revision C.01) Gaussian Inc., Wallingford, CT, **2016**.

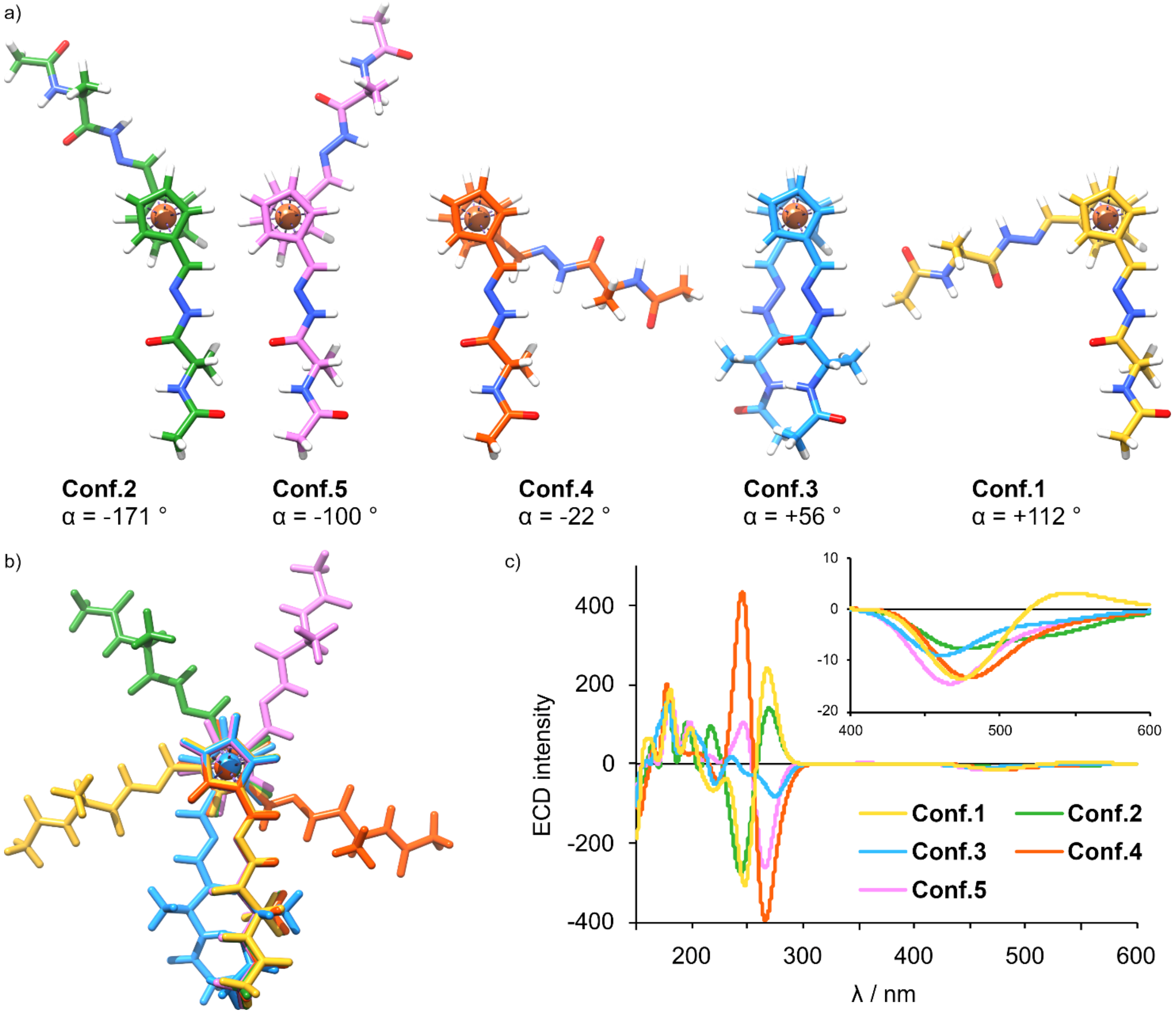

Figure S21. Geometry-optimized conformers of **Fc(L-1)$_2$** model: a) models of **Conf.1** - **Conf.5**; b) superposition of **Conf.1** - **Conf.5**; c) comparison of theoretical ECD spectra for **Conf.1** - **Conf.5** (without normalization).

Fc(L-1)$_2$ (model)

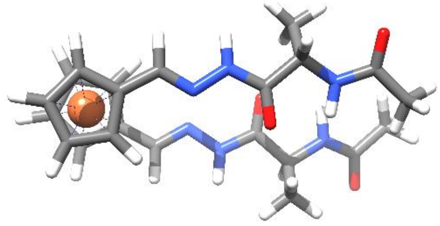

Electronic Energy = -2746.135580 Hartree
Zero-point Energy Correction = 0.487651 Hartree
Thermal Correction to Energy = 0.520259 Hartree
Thermal Correction to Enthalpy = 0.521203 Hartree
Thermal Correction to Free Energy = 0.423987 Hartree
Number of imaginary frequencies: 0

Symbol X Y Z
C 6.13744300 1.57513800 0.80900300
C 5.03018000 2.28368700 0.07749300
O 5.20149400 3.34705500 -0.49705700
N 3.82415500 1.65502300 0.11096100
H 3.68324800 0.88338000 0.76296800
C 2.61864500 2.29203000 -0.36637000
H 2.64288800 3.36002900 -0.06250400
C 2.49724100 2.23473100 -1.88268200
C 1.45347900 1.66125200 0.39351600
H 2.35192000 1.19794000 -2.22143200
H 1.66472400 2.85183400 -2.25752300
H 3.41741600 2.64192400 -2.32665500
O 1.62393400 0.93707100 1.35566500
N 0.22208200 2.01835700 -0.06924600
N -0.90185900 1.59911300 0.55172700
C -2.01414900 1.97742600 0.04776000
H -2.05487600 2.61672000 -0.85655400
C -3.28769600 1.58516500 0.62687500
C -3.50173800 0.66973300 1.70901600
C -4.57300900 1.97974900 0.12801000
C -4.90565300 0.51457300 1.87874200
H -2.71244800 0.17504200 2.27816700
C -5.56690200 1.31998800 0.90347600
H -4.74598400 2.65906100 -0.70930700
H -6.64593600 1.40140700 0.76487900
H -5.39280800 -0.12971200 2.61231500
H 0.14249200 2.61630700 -0.89687800
Fe -4.33644000 -0.03698700 -0.01038000
C 6.21775000 -1.67307100 -0.87613000
C 5.09441600 -2.31593200 -0.11002000
O 5.22920100 -3.37796300 0.47676000
N 3.91849900 -1.63116800 -0.12102500
H 3.79255600 -0.85869300 -0.77538500
C 2.70295000 -2.20754600 0.40467500
H 2.68445500 -3.28551200 0.14006800
C 2.61932700 -2.09238400 1.92057400
C 1.53908800 -1.56174900 -0.34391900
H 2.51738300 -1.04015600 2.22773900
H 1.77200400 -2.66317100 2.33394200
H 3.53402600 -2.51816000 2.35861900
O 1.70876700 -0.81718600 -1.29064500
N 0.30980600 -1.93397800 0.11205200
N -0.81776200 -1.53573100 -0.51548900
C -1.92404800 -1.95840300 -0.03334000
H -1.95460600 -2.61300600 0.86063300
C -3.20250200 -1.60956400 -0.62900400
C -3.43640500 -0.70150800 -1.71327300
C -4.47859200 -2.06252800 -0.15632600
C -4.84226300 -0.60943200 -1.91078200
H -2.65959300 -0.17043700 -2.26665400
C -5.48567800 -1.44639400 -0.95033800

H -4.63715600 -2.75209500 0.67541200
H -6.56250800 -1.57698900 -0.83387800
H -5.34319200 0.01460200 -2.65255500
H 0.23610900 -2.54542500 0.93057300
H 5.86226000 0.56627400 1.15140200
H 7.02341500 1.51648000 0.15896900
H 6.42292300 2.17891700 1.68546300
H 5.96777300 -0.68012900 -1.27841000
H 7.10101500 -1.59322800 -0.22418300
H 6.49701400 -2.33442500 -1.71174000

[**Fc(L-1)$_2$**]$^+$ (model)

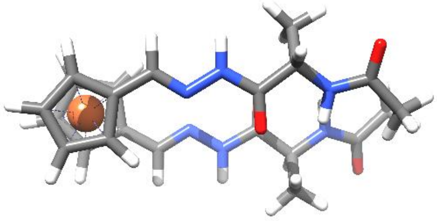

Electronic Energy = -2745.960078 Hartree

Zero-point Energy Correction = 0.487334 Hartree

Thermal Correction to Energy = 0.520506 Hartree

Thermal Correction to Enthalpy = 0.521450 Hartree

Thermal Correction to Free Energy = 0.422667 Hartree

Number of imaginary frequencies: 0

Symbol	X	Y	Z
C	6.01251800	1.46962100	0.96297700
C	4.97739800	2.20664500	0.15894600
O	5.22670500	3.22575400	-0.46532700
N	3.73362400	1.65391900	0.18971400
H	3.53504300	0.93343400	0.88294900
C	2.58032400	2.31758200	-0.37136300
H	2.61471300	3.39347800	-0.09573100
C	2.54294900	2.21836800	-1.89076000
C	1.35651800	1.74343600	0.33123000
H	2.39961300	1.17490200	-2.20887700
H	1.74725100	2.83968200	-2.33218000
H	3.49572600	2.59448300	-2.29027600
O	1.43378400	1.03311500	1.31235400
N	0.15747700	2.12332600	-0.21520300
N	-0.99011000	1.73798900	0.35723200
C	-2.08623100	2.09141400	-0.19619100
H	-2.12057900	2.68671200	-1.12711200
C	-3.35270600	1.70688200	0.40857100
C	-3.53077500	0.95497500	1.62010500
C	-4.65227300	1.97447100	-0.12465300
C	-4.92021300	0.78461300	1.83379100
H	-2.72321600	0.56696200	2.24364500
C	-5.60825600	1.41803800	0.76642500
H	-4.86222500	2.51434600	-1.04916400
H	-6.68891600	1.41440400	0.61643300
H	-5.38007000	0.23445100	2.65483800
H	0.13501300	2.71766200	-1.04964600
Fe	-4.31592600	-0.07936700	-0.01491800
C	6.23381000	-1.61082000	-1.01115800
C	5.13493400	-2.25865300	-0.21680200
O	5.28356100	-3.32669200	0.35786100
N	3.95882700	-1.57835100	-0.18768700
H	3.83586600	-0.74017600	-0.75402100
C	2.78543500	-2.11818500	0.45313100
H	2.77351600	-3.21373000	0.28020500
C	2.78647800	-1.87831300	1.95981800
C	1.56479100	-1.55022700	-0.25553400
H	2.69366600	-0.80511100	2.18990700
H	1.96664100	-2.41129000	2.46786000
H	3.72935000	-2.26255700	2.37684300
O	1.63403100	-0.79803300	-1.20292300
N	0.36652700	-1.98759000	0.26168800
N	-0.78568200	-1.63957500	-0.31551600
C	-1.86949500	-2.10531300	0.18294000
H	-1.87772300	-2.75969700	1.07353700
C	-3.14364800	-1.80729400	-0.44969900
C	-3.35100500	-0.98839100	-1.60829900
C	-4.41981500	-2.20787400	0.04025400
C	-4.75123100	-0.92313000	-1.84657400
H	-2.56148700	-0.51558000	-2.19432700
C	-5.40751800	-1.67663300	-0.82407600

H -4.59527100 -2.80120100 0.93877500
H -6.48548500 -1.78894900 -0.70108400
H -5.23652300 -0.38382700 -2.66046400
H 0.35926900 -2.63301700 1.05863800
H 5.71131300 0.44078800 1.21467400
H 6.96238000 1.45616100 0.40903400
H 6.19101200 2.01983800 1.90142300
H 5.98785900 -0.59746600 -1.36161100
H 7.14697700 -1.57575200 -0.39794300
H 6.45912400 -2.24384800 -1.88425300

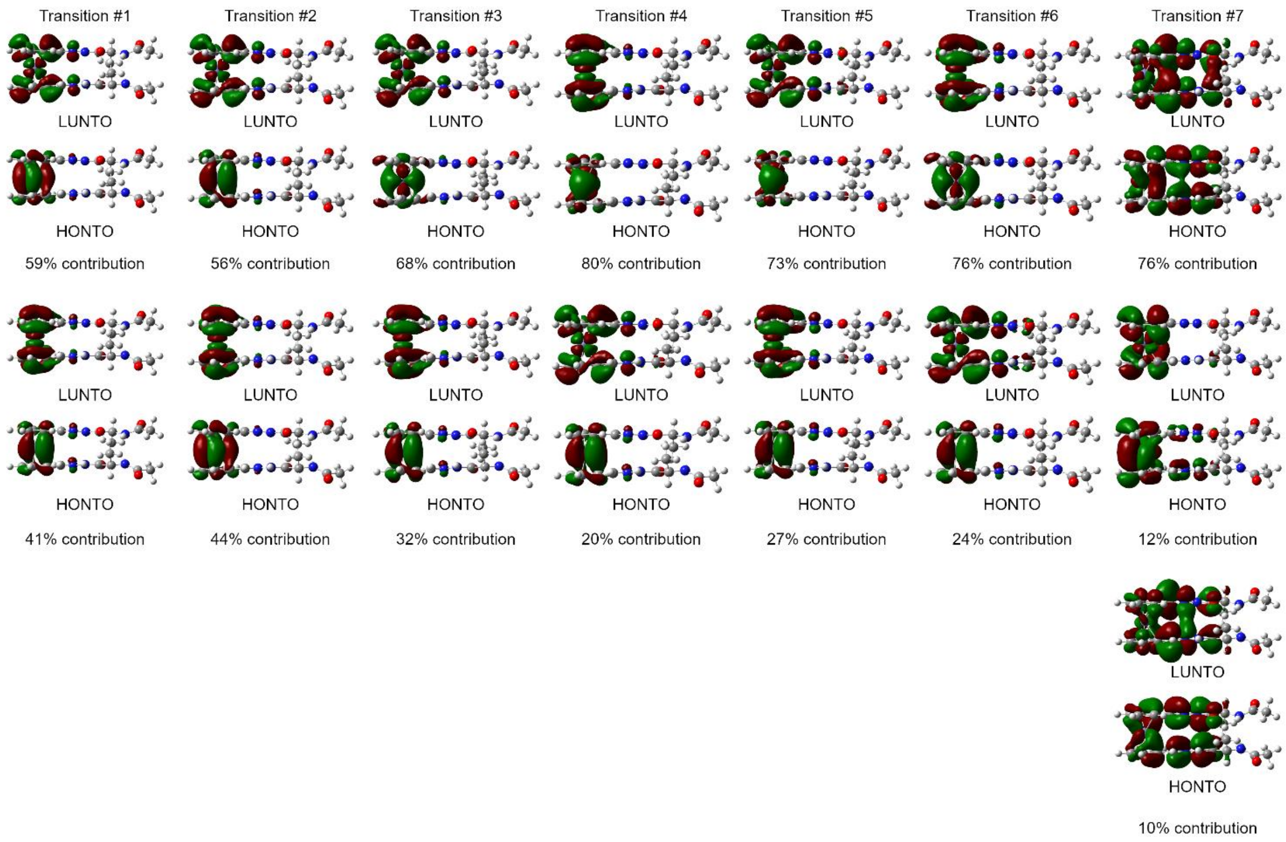

Figure S22. NTOs calculated for the seven lowest-energy transitions (500-300 nm) in the theoretical spectrum of **Fc(L-1)$_2$**

Electrochemistry

For electrochemical measurements, a three-electrode cell containing a glassy carbon electrode (GCE, Ø = 3 mm, Mineral, Poland) as the working electrode, Ag/$AgNO_3$ (10 mM $AgNO_3$ in MeCN) as the reference (BASi, USA), and platinum wire as the counter electrode (MINERAL, Poland) was employed. Prior to each measurement, the GCE was polished to a mirror-like finish using 0.05 µm alumina suspension on a polishing cloth (Buehler). An electrolytic bridge filled with 0.1 M Bu_4NPF_6 in DCM separated the reference electrode from the working solution. All electrochemical measurements were carried out in 0.1 M Bu_4NPF_6 in DCM at room temperature under an argon atmosphere using a CHI 1030 potentiostat (CH Instruments, USA). Cyclic voltammetry (CV) measurements were performed at scan rates ranging from 10 to 500 mV/s.

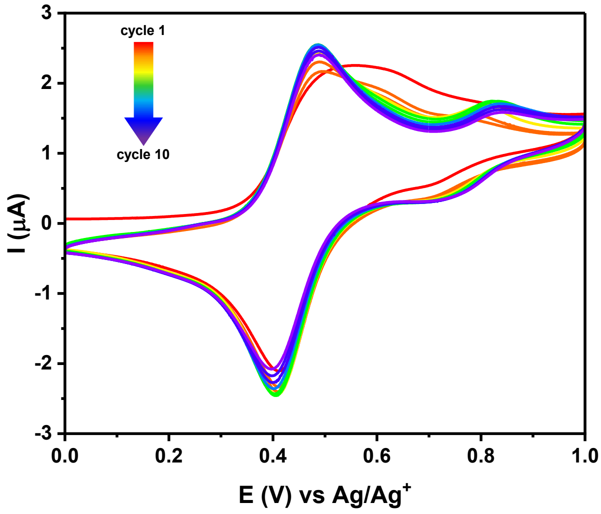

Figure S23. Cyclic voltammograms of 0.1 mM **Fc_3(L-2)$_2$**recorded in DCM containing 100 mM Bu_4NPF_6 at scan rate 10 mV/s (first 10 cycles).

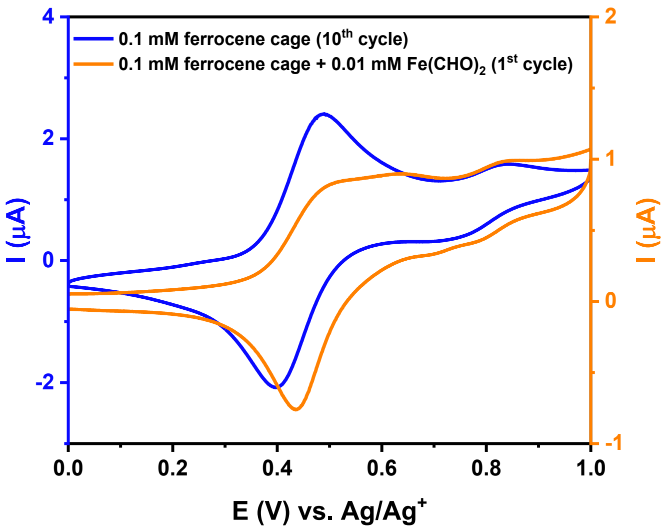

Figure S24. Comparison of cyclic voltammograms of 0.1 mM **Fc₃(L-2)₂** and mixture of 0.1 mM ferrocene with dialdehyde ferrocene (molar ratio 10:1) recorded in DCM containing 100 mM Bu_4NPF_6 at scan rate 10 mV/s (10th cycle for the ferrocene cage and 1st cycle for the mixture).

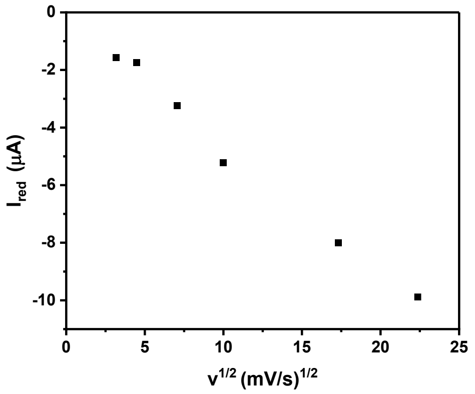

Figure S25. Dependence of the cathodic peak current at 0.40 V on the square root of the scan rate.

Table S2. Electrochemical potential and current values related to the redox processes for **Fc_3(L-2)$_2$** and derivatives constituting the structure of the ferrocene cage, calculated based on CV curves at 10th cycle (scan rate 10 mV/s).

	E_{ox} (V)	E_{red} (V)	E_f (V)	ΔE (mV)	I_{ox} (µA)	I_{red} (µA)	I_{red}/I_{ox}
**Fc_3(L-2)$_2$**	0.490	0.397	0.444	93	2.134	-2.341	1.10
**Fc_3(L-2)$_2$**	0.845	0.742	0.794	103	0.27	-0.35	1.30
**Fc(CHO)$_2$**	0.845	0.777	0.811	68	2.177	-1.778	0.82
**Fc(L-1)$_2$**	0.467	0.403	0.435	64	7.903	-7.368	0.93
**L-1**	0.807	-	-	-	0.03723	-	-
**L-2**	0.685	-	-	-	0.07782	-	-
